# Specificity of resistance and geographic patterns of virulence in a vertebrate host-parasite system

**DOI:** 10.1186/s12862-019-1406-3

**Published:** 2019-03-19

**Authors:** Agnes Piecyk, Olivia Roth, Martin Kalbe

**Affiliations:** 1 Department of Evolutionary Ecology, Max Planck Institute for Evolutionary Biology, August-Thienemann-Straße 2, 24306 Plön, Germany; 2 Marine Evolutionary Ecology, GEOMAR Helmholtz Centre for Ocean Research Kiel, Düsternbrookerweg 20, 24105 Kiel, Germany

**Keywords:** Host-parasite interaction, Immunological heterogeneity, Virulence, Stickleback, *Schistocephalus solidus*

## Abstract

**Background:**

Host genotype - parasite genotype co-evolutionary dynamics are influenced by local biotic and abiotic environmental conditions. This results in spatially heterogeneous selection among host populations. How such heterogeneous selection influences host resistance, parasite infectivity and virulence remains largely unknown. We hypothesized that different co-evolutionary trajectories of a vertebrate host-parasite association result in specific virulence patterns when assessed on a large geographic scale. We used two reference host populations of three-spined sticklebacks and nine strains of their specific cestode parasite *Schistocephalus solidus* from across the Northern Hemisphere for controlled infection experiments. Host and parasite effects on infection phenotypes including host immune gene expression were determined.

**Results:**

*S. solidus* strains grew generally larger in hosts coming from a population with high parasite diversity and low *S. solidus* prevalence (DE hosts). Hosts from a population with low parasite diversity and high *S. solidus* prevalence (NO hosts) were better able to control the parasite’s growth, regardless of the origin of the parasite. Host condition and immunological parameters converged upon infection and parasite growth showed the same geographic pattern in both host types.

**Conclusion:**

Our results suggest that NO sticklebacks evolved resistance against a variety of *S. solidus* strains, whereas DE sticklebacks are less resistant against *S. solidus*. Our data provide evidence that differences in parasite prevalence can cause immunological heterogeneity and that parasite size, a proxy for virulence and resistance, is, on a geographic scale, determined by main effects of the host and the parasite and less by an interaction of both genotypes.

**Electronic supplementary material:**

The online version of this article (10.1186/s12862-019-1406-3) contains supplementary material, which is available to authorized users.

## Background

The interaction of an organism with its environment is a hallmark of life and a pre-requisite for natural selection. Local adaptation is driven by abiotic conditions and biotic interactions within and between species. Among the strongest evolutionary processes is the co-evolution between hosts and parasites [1–5]. Parasites rely on host resources and have the potential to drastically reduce host fitness [6]. To diminish the harm of parasites, effective defence strategies have evolved on the host side [4, 7]. However, heterogeneous environments select for different defence strategies among host populations, which results in immunological heterogeneity [8, 9]. The variation of host defence against parasites can range from mechanisms that decrease the risk of infection to processes that diminish the harm of parasites, such as resistance (i.e. the prevention of infection or the control of parasite growth) and tolerance (i.e. the ability to limit health or fitness effects of a distinct infection intensity) [10, 11]. Likewise, parasite infectivity and virulence (i.e. the detrimental effects on host traits related to fitness) are spatially structured both by environmental parameters and co-evolutionary processes.

**Fig. 1:**
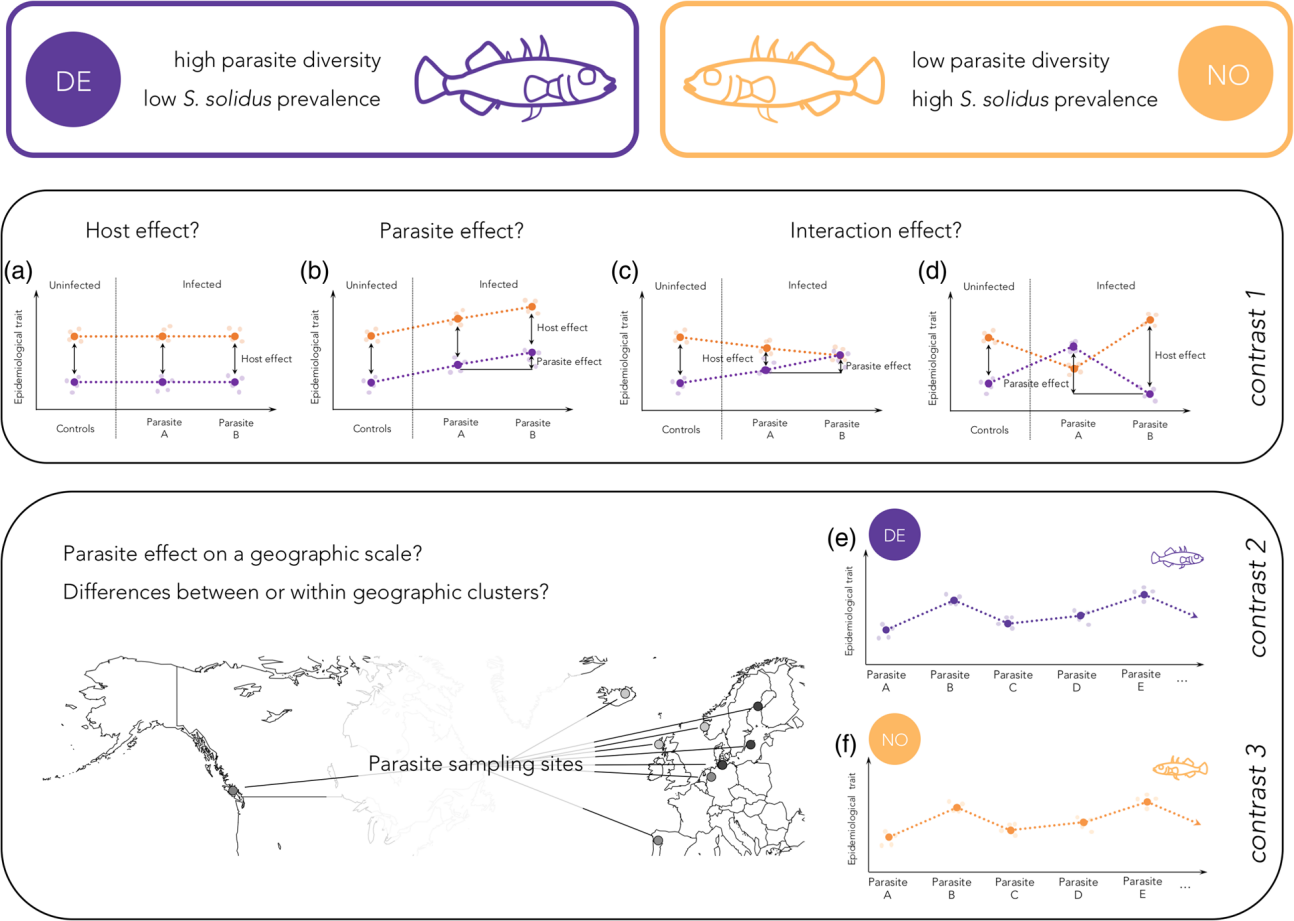
Theoretical framework of the study. Reference hosts came from two contrasting populations, indicated by violet (DE) and orange (NO) dots and lines. For the sake of simplicity, we exemplify possible outcomes with a subset of hypothetical parasites A to E. We asked whether main effects of the host, the parasite, and/or host-parasite interaction effects shaped epidemiological traits (life history traits of the host and/or the parasite). (**a**, **b**, **c**, **d**) Partitioning of host, parasite, and interaction effects on an epidemiological trait. (**a**, **b**) Host genotype and parasite genotype main effects. The host effect (vertical spacing between the two lines) indicates the genetic difference between the two host types. Parallel horizontal lines in (**a**) indicate absence of a plastic response towards infection. Differences among hosts that are infected with the same parasite (vertical spacing between the dots) indicate a phenotypic plastic response of the parasite. The positive slope in (**b**) indicates different effects of the two parasite types (parasite effect) and thus a phenotypic plastic response of the host and the parasite. (**c**) and (**d**) demonstrate host genotype-parasite genotype interaction effects, because the host effect depends on the parasite type. Crossing reaction norms in (**d**) clearly show the interaction effect; but note in (**c**) that the main-effect components can cumulate, causing non-crossing reaction norms. We tested the predictions with data from *contrast 1*. (**e**, **f**) To further understand the parasite effect on a larger geographic scale, each of the two host types was exposed to parasites from different geographic clusters across the Northern Hemisphere. We tested these predictions with data from *contrast 2* and *contrast 3*

The epidemiological traits are shaped through main effects of the host and the parasite and by interaction effects (Fig. 1). The relative contribution of each of the interaction partners may differ along the infection process and depends on the geographic scale and the degree of environmental heterogeneity. Controlled infection experiments can be used to first identify environmental and evolutionary causes shaping the epidemiological traits and, second, to study the mechanisms and the adaptive significance thereof. Experiments revealed rapid and adaptive co-evolution of host and parasite genotypes in various systems, including phage-bacteria associations [4, 12], malaria systems [13, 14], plant-pathogen interactions [15, 16], and immune gene evolution in three-spined sticklebacks [17, 18]. We chose the association of three-spined sticklebacks and their specific macroparasite *Schistocephalus solidus* to determine host and parasite effects along the infection process and on different geographic scales.

Three-spined sticklebacks (*Gasterosteus aculeatus*; hereafter ‘sticklebacks’) live in numerous freshwater and marine habitats across the Northern Hemisphere. Various studies reported habitat-specific immune responses [19–24]. A lot of attention has been paid on the “supermodel” [25] interaction between sticklebacks and the cestode *Schistocephalus solidus*, as both can be bred in the laboratory facilitating controlled infection experiments [25]. *S. solidus* has a three-host life cycle with copepods as first intermediate host and *G. aculeatus* as specific second intermediate host. *S. solidus* grows massively in the body cavity of the fish, sometimes even exceeding the host’s weight [26, 27]. Reproduction is confined to the definite host, mostly piscivorous birds. The parasite’s reproductive output is directly related to its size [28]. *S. solidus’* detrimental effects on sticklebacks were shown both in nature and in the laboratory and have been linked to the size of the parasite [27–30]. This cestode is assumed to be a driving force of divergent selection in three-spined sticklebacks [31]. Studies using hosts and parasites from different populations from Europe [32], from across continents [33] and in vitro leukocyte responses [34] indicate local adaptation of sticklebacks and *S. solidus*. It was suggested that *S. solidus* growth depends on host and/or parasite population-specific traits [35].

**Fig. 2:**
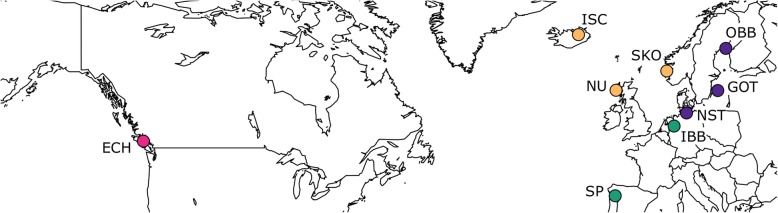
Sampling sites. Sticklebacks originated from Lake Großer Plöner See, Germany (DE), ~ 25 km from Neustädter Binnenwasser (NST; one of the sampling sites of *S. solidus*) and Lake Skogseidvatnet, Norway (NO). *S. solidus* were sampled from nine different locations across Europe and the Pacific (more information in Additional file 1: Table S1). Colors indicate four geographic clusters (pink: Pacific, orange: Atlantic, violet: Baltic, green: European Inland). The map was drawn with the R package *maps* [77]; colors were chosen from the ColorBrewer palette [76]

We assumed that sticklebacks evolved environment-specific immunological adaptations to *S. solidus* and that *S. solidus* evolved environment-specific virulence. We specifically asked if such divergent evolution could cause different immunological activation in response to a variety of *S. solidus* strains (i.e. *S. solidus* parasites from distinct locations). The following was hypothesized: (i) the infection phenotype differs between sticklebacks from heterogeneous environments (indicating a host effect); (ii) the infection phenotype differs between *S. solidus* strains (indicating a parasite effect); (iii) the infection phenotype differs according to stickleback-*S. solidus* interactions (indicating an interaction effect) (Fig. 1). These hypotheses were tested with three distinct analyses. First, hosts from two contrasting reference populations of *G. aculeatus* were experimentally infected with *S. solidus* from four European locations in order to test if host effects, parasite effects and/or interaction effects influenced *S. solidus* infection phenotypes in *G. aculeatus* (the corresponding analyses are referred to as *contrast 1*; Fig. 1). In order to test the parasite effect in further detail, each of these reference host types was infected with *S. solidus* strains from across the Northern Hemisphere (the corresponding analyses are referred to as *contrast 2* and *contrast 3*; Fig. 1; Table 1; Additional file 1: Table S1). *S. solidus* sampling sites covered four geographic areas (clustered localities) corresponding to *G. aculeatus* phylogeny: the Atlantic region (NU, ISC, SKO), the Baltic region (OBB, NST, GOT), European Inland (SP, IBB), and the Pacific (ECH) (Fig. 2; Table 1).

**Table 1:** Summary table of sample sizes within contrasts of interest

		Baltic						European Inland				Pacific		Atlantic						
Analysis	Host	OBB		GOT		NST		SP		IBB		ECH		NU		ISC		SKO		control
*contrast 1*	DE (A)	na		na		(a)	2	na		(a)	5	na		na		(a)	2	(d)	4	20
	DE (B)	na		na		(b)	10	na		(b)	3	na		na		(b)	5	(a)	7	18
	DE (C)	na		na		(c)	8	na		(c)	10	na		na		(c)	5	(c)	3	20
	NO (A)	na		na		(a)	2	na		(a)	3	na		na		(a)	1	(d)	4	20
	NO (B)	na		na		(b)	4	na		(b)	2	na		na		(b)	6	(a)	8	20
	NO (C)	na		na		(c)	2	na		(c)	2	na		na		(c)	5	(c)	2	20
*contrast 2*	DE (D)	(a)	4	(a)	5	(a)	4	(a)	1	na		(a)	3	(a)	1	na		(a)	7	20
	DE (E)	(b)	2	(b)	0	(b)	3	(b)	0	na		(b)	2	(b)	3	na		(b)	0	20
	DE (F)	(c)	4	(c)	3	(c)	9	(c)	7	na		(c)	2	(c)	6	na		(c)	5	20
*contrast 3*	NO (A)	(a)	1	(d)	1	(a)	2	(a)	2	(a)	3	(a)	1	(b)	5	(a)	1	(d)	4	20
	NO (B)	(b)	10	(b)	4	(b)	4	(d)	5	(b)	2	(c)	4	(d)	7	(b)	6	(a)	8	20
	NO (C)	(c)	1	(a)	2	(c)	2	(b)	2	(c)	2	(b)	0	(c)	6	(c)	5	(c)	2	20

Naïve laboratory bred first generation offspring from three-spined sticklebacks *Gasterosteus aculeatus* from lake Großer Plöner See, Germany (DE), and Lake Skogseidvatnet, Norway (NO), were infected with *Schistocephalus solidus* parasites from different geographic locations or sham-exposed as controls. The top row indicates *S. solidus* geographic cluster; abbreviations in the second row refer to *S. solidus* sampling sites (OBB: Obbola, Sweden; GOT: Gotland, Sweden; NST: Neustädter Binnenwasser, Germany; SP: Xinzo de Limia, Spain; IBB: Ibbenbürener Aa, Germany; ECH: Vancouver Island, Canada; NU: North Uist, Scotland; ISC: Lake Myvatn, Iceland; SKO: Lake Skogseidvatnet, Norway; control: sham-exposed control). Capital letters indicate fish families (offspring of one pair of sticklebacks), lower case letters indicate worm sibships (offspring of one pair of worms). Per treatment, i.e. fish family x worm sibship combination, 100 copepods and subsequently 20 fish were exposed to single infective *S. solidus* larvae or sham-exposed; combinations with ‘na’ were not included in the respective analysis. Numbers in columns of *S. solidus* exposed fish indicate the number of infected individuals. We used *contrast 1* to test for host, parasite and interaction effects; *contrast 2* and *contrast 3* were used to test parasite effects on a broader geographic scale. NO data in *contrast 1* is a data subset of *contrast 3*. We accounted for multiple testing

The two host populations differ remarkably in parasite diversity (Shannon diversity index) and abundance (the mean number of parasites per fish) [24, 36]. Parasite diversity is high and *S. solidus* prevalence (the number of infected individuals) is low (< 1%) in the German habitat (DE), whereas *S. solidus* prevalence is high and parasite diversity is low in the Norwegian population (NO) (20 to > 50%). Under the assumption that immune defence is costly and co-evolves with parasite virulence [7, 37–40], we hypothesized that sticklebacks from the highly *S. solidus* exposed (NO) population evolved *S. solidus* specific resistance, whereas this might not be the case for the rarely *S. solidus* exposed (DE) population. We suggested that *S. solidus* specific resistance could be effective against sympatric and potentially even allopatric strains. In order to cover numerous important parameters along the infection process, infection rates and the size of the parasite, as well as host condition and immunological parameters were determined [10]. The size of the parasite is used a measure of host resistance and parasite virulence [11, 32, 41]. The immunological activation was inferred from the size of the major immune organs and by immune gene expression analyses. We asked whether host population and/or parasite strain, cluster or growth caused distinct gene expression profiles. This study investigates evolutionary and proximate (physiological and molecular) causes of immunological heterogeneity, the specificity of resistance and the contribution of host and parasite on infection phenotypes.

## Results

Both intermediate hosts (copepods and sticklebacks) were infected with *S. solidus* from every location (Additional file 1: SI.1; Tables S2 and S3). We obtained 227 plerocercoids from 1342 fish (excluding two infected controls and one double infected fish). The average weight of *S. solidus* plerocercoids 55 (+/− 2) days post exposure (DPE) was 61.8 mg and varied between 0.6 mg and 151.4 mg. Neither infection rates in copepods nor infection rates in fish influenced *S. solidus* size in the fish (LMMs for average parasite index (PI) per worm sibship as dependent variable; worm origin, infection rates in copepods and in fish as fixed effects, round as random term).

*Contrast 1*, the comparison of DE and NO hosts infected with four different European *S. solidus* strains, included 587 fish: 118 controls (excluding two infected DE controls), 105 infected fish, 364 exposed but uninfected fish; 11 fish died. *Contrast 2*, testing the parasite effect in DE hosts, included 522 fish: 60 controls, 71 infected fish, 335 exposed but uninfected fish; 14 fish died. *Contrast 3*, testing the parasite effect in NO hosts, included 60 controls, 92 infected fish, 433 exposed but uninfected fish; 15 fish died.

### Constitutive differences between the host populations (contrast 1)

**Fig. 3:**
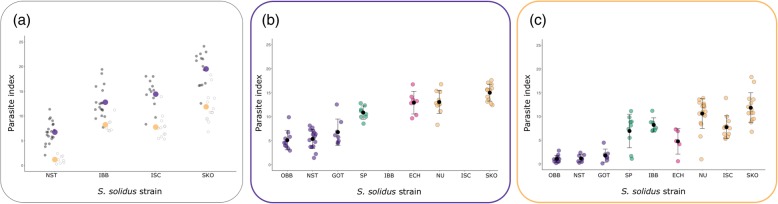
*S. solidus* growth differs significantly between host populations and between geographically clustered parasite strains. Naïve laboratory bred F1 offspring from sticklebacks from lake Großer Plöner See, Germany (DE), and Lake Skogseidvatnet, Norway (NO), were experimentally infected with single *S. solidus* larvae from nine different locations (‘strains’) across the Northern Hemisphere. Fish were dissected 55 (+/− 2) days after exposure to the parasite. The parasite index (PI) was calculated as the proportion of the parasite’s weight from the total weight of infected fish. (**a**) DE and NO hosts were infected with four different European *S. solidus* strains (*contrast 1*). Black and white dots represent individuals; violet: mean parasite indices in DE hosts; orange: mean parasite indices in NO hosts (Additional file 1: Table S6). (**b**) Parasite indices in DE hosts (*contrast 2*). Black dots and bars indicate the mean and the standard deviation. Color coding follows Fig. 2. (**c**) Parasite indices in NO hosts (*contrast 3*). Black dots and bars indicate the mean and the standard deviation. Color coding follows Fig. 2

*Contrast 1*, the combination of the two hosts and four *S. solidus* strains, was used to test for host effects, parasite effects and host-parasite interaction effects on infection rates and infection phenotypes (Fig. 1; Table 1). *S. solidus* infection rates were consistent among host populations (host effect: Χ^2^_1_ = 2.27, *p* = 0.132; *S. solidus* effect: Χ^2^_3_ = 0.882, *p* = 0.830; host-parasite interaction effect: Χ^2^_3_ = 6.42, *p* = 0.093; Additional file 1: Table S4). However, all four *S. solidus* strains were significantly smaller in NO hosts (parasite index, PI, the relative weight of *S. solidus* in the host [27]; host effect: F_1,95_ = 23.48, *p* < 0.0001). The differences between *S. solidus* strains were independent of the host population (host-parasite interaction effect on PI: F_3,95_ = 0.995, *p* = 0.399) (Fig. 3; Additional file 1: Tables S5-S7).

We detected constitutive differences in condition and immunological parameters of the two stickleback populations (more information in Additional file 1: SI.3). DE sticklebacks had a significantly higher condition (CF; an estimate of the overall condition [42]) if they were uninfected (Χ^2^_1_ = 44.252, *p* < 0.0001) or infected with *S. solidus* from the Baltic (NST) (Χ^2^_1_ = 10.48, *p* = 0.001). Hepatosomatic indices (HSI, an estimate of metabolic reserves [43]) were higher in DE controls compared to NO controls (Χ^2^_1_ = 26.93, *p* < 0.0001). Head kidney indices (HKI, the relative weight of the major immune organ in fish) were generally higher in DE fish (Χ^2^_4_ = 49.47, *p* < 0.0001) and DE controls showed higher reactive oxygen species (ROS) production of head kidney leukocytes (Χ^2^_1_ = 24.1, *p* < 0.0001). Splenosomatic indices (SSI, the relative weight of the major secondary immune organ [44]) were significantly higher in DE controls (Χ^2^_1_ = 79.38, *p* < 0.0001) and in DE hosts infected with Baltic (NST) *S. solidus* (Χ^2^_1_ = 30.75, *p* < 0.0001) or European Inland (IBB) *S. solidus* (Χ^2^_1_ = 19.02, *p* < 0.0001). The effects were not directly related to *S. solidus* size but to *S. solidus* strain. We detected no significant differences in these condition and immunological parameters between DE and NO sticklebacks if they were infected with *S. solidus* from two Atlantic populations (SKO, ISC) (Additional file 1: Figure S1).

Total RNA from spleen was used to determine expression levels of 24 key immune genes. We ran non-parametric permutational multivariate analyses of variance (PERMANOVA) including host and parasite main effects and their interaction. The main effects were significant predictors while the interaction did not influence immune gene expression profiles (host effect: PERMANOVA_*innate*_: F_1,148_ = 10.69, *p* < 0.0001; PERMANOVA_*adaptive*_: F_1,148_ = 13.58, *p* < 0.0001; PERMANOVA_*complement*_: F_1,148_ = 7.03, *p* = 0.0001; *S. solidus* effect: PERMANOVA_*innate*_: F_4,148_ = 3.74, *p* = 0.0002; PERMANOVA_*adaptive*_: F_4,148_ = 2.73, *p* = 0.007; PERMANOVA_*complement*_: F_4,148_ = 3.82, *p* = 0.0002; host-parasite interaction effect: PERMANOVA_*innate*_: F_4,148_ = 0.93, *p* = 0.45; PERMANOVA_*adaptive*_: F_4,148_ = 1.01, *p* = 0.41; PERMANOVA_*complement*_: F_4,148_ = 0.40, *p* = 0.94). Pairwise PERMANOVAs were used a posteriori in order to identify significantly different groups [45].

**Fig. 4:**
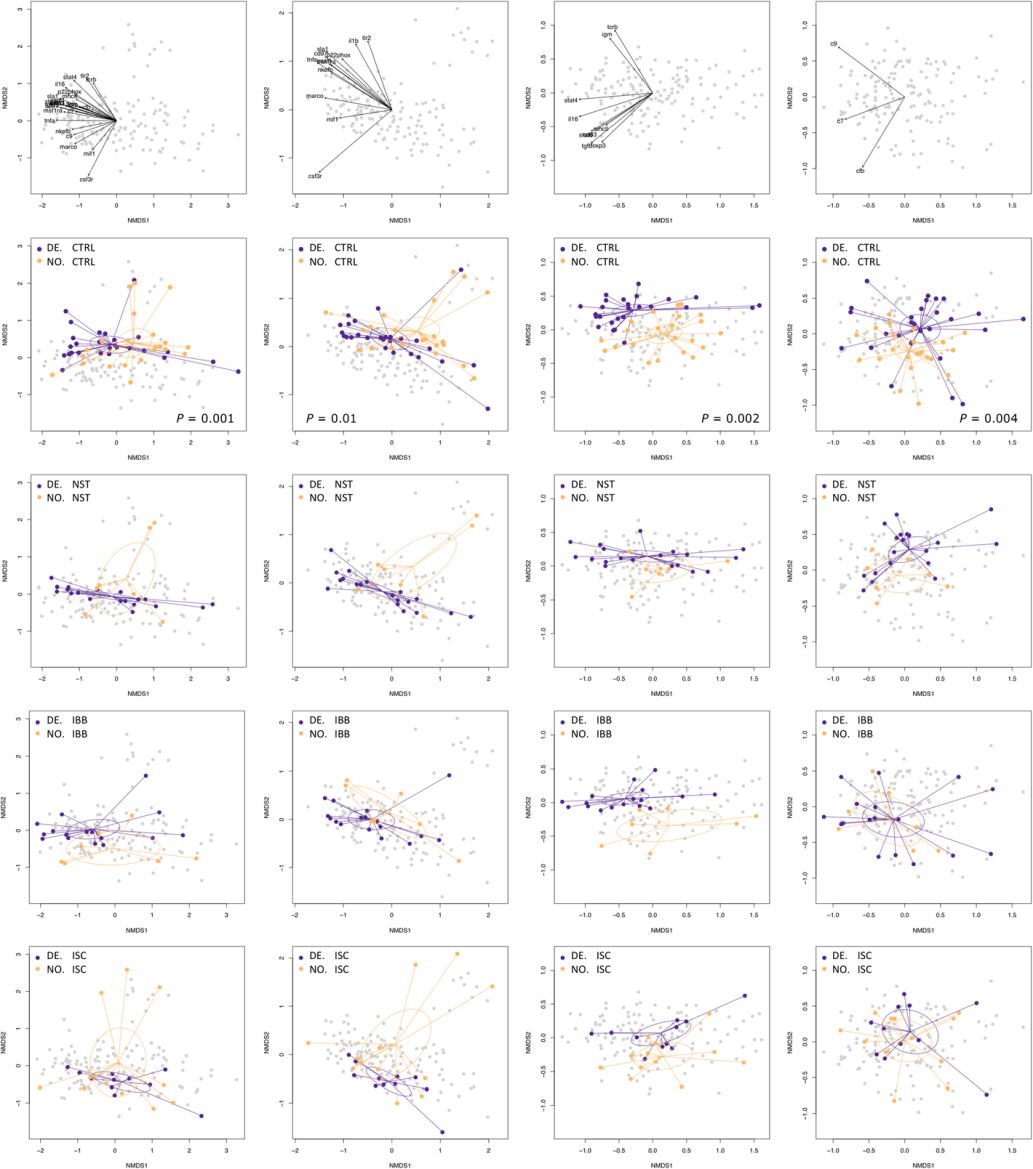
Multivariate gene expression patterns differ between DE and NO sticklebacks. Non-metric multidimensional scaling (NMDS) plots on Euclidian distances and two dimensions comparing data from NO and DE sticklebacks (*contrast 1*). NMDS were based on log10-transformed calibrated normalized relative quantities (CNRQ values) of all 24 immune genes, twelve genes of innate immunity (*marco*, *mst1ra*, *mif*, *il-1β*, *tnfr1*, *saal1***,** *tlr2, csf3r*, *p22*^*phox*^*, nkef-b, sla1, cd97*), nine genes of adaptive immunity (*stat4*, *stat6*, *igm*, *cd83*, *foxp3*, *tgf-β, il-16, mhcII, tcr-β*), or three genes of the complement system (*cfb, c7, c9*). Each dot represents one individual; colors refer to the host population. Ellipses represent 95% confidence intervals. *P*-values are shown if significant after FDR-correction. The contribution of each gene is shown in the first row. The second row shows data from sham-exposed (CTRL) sticklebacks. The third to sixth row show data from infected individuals. Function metaMDS() was used to plot the NMDS; the contribution of each gene was plotted by use of the envfit() function (both functions are implemented in R package *vegan* [74])

Immune gene expression profiles differed significantly between DE and NO controls (PERMANOVA_*innate*_: F_1,48_ = 3.32, *p* < 0.001; PERMANOVA_*adaptive*_: F_1,48_ = 6.76, *p* = 0.002; PERMANOVA_*complement*_: F_1,48_ = 4.78, *p* = 0.004; Additional file 1: Table S11; Fig. 4). DE sticklebacks had higher expression levels of genes of innate and adaptive immunity, while complement genes were lower expressed than in NO sticklebacks (Additional file 1: Table S8; Figure S6). ISC *S. solidus* infection caused different innate immune gene expression in DE and NO sticklebacks (PERMANOVA_*innate*_: F_1,22_ = 3.58, *p* = 0.004; Additional file 1: Table S9; Fig. 4), which was driven by remarkably low expression of Interleukin-1β (*il-1β*) in DE sticklebacks (F_1,18_ = 20.0, *p* < 0.001) (Additional file 1: Table S9; Figure S6). Expression profiles of NST-, IBB- and SKO-infected fish did not differ significantly between host populations (Fig. 4).

### Parasite indices show a geographic pattern in both host types

To further understand the effect of the parasite on infection phenotypes, we exposed DE hosts (*contrast 2*) and NO hosts (*contrast 3*) to *S. solidus* strains from across the Northern Hemisphere (Fig. 1). The infection rates did not differ significantly between parasite strains in DE sticklebacks (*contrast 2*: Χ^2^_6_ = 7.15, *p* = 0.307), but did so in NO sticklebacks (*contrast 3*: Χ^2^_8_ = 21.62, *p* = 0.006) (Additional file 1: Tables S3–S4). Parasite indices differed between parasite strains (*contrast 2*: F_6,62_ = 42.39, *p* < 0.0001; *contrast 3*: F_8,81_ = 61.09, *p* < 0.0001). We found a clear pattern with *S. solidus* from the Baltic being significantly smaller than worms from the other origins; Atlantic *S. solidus* were the largest in both host types (Additional file 1: Tables S10-S12; Fig. 3).

### Immune gene expression is parasite strain specific

**Fig. 5:**
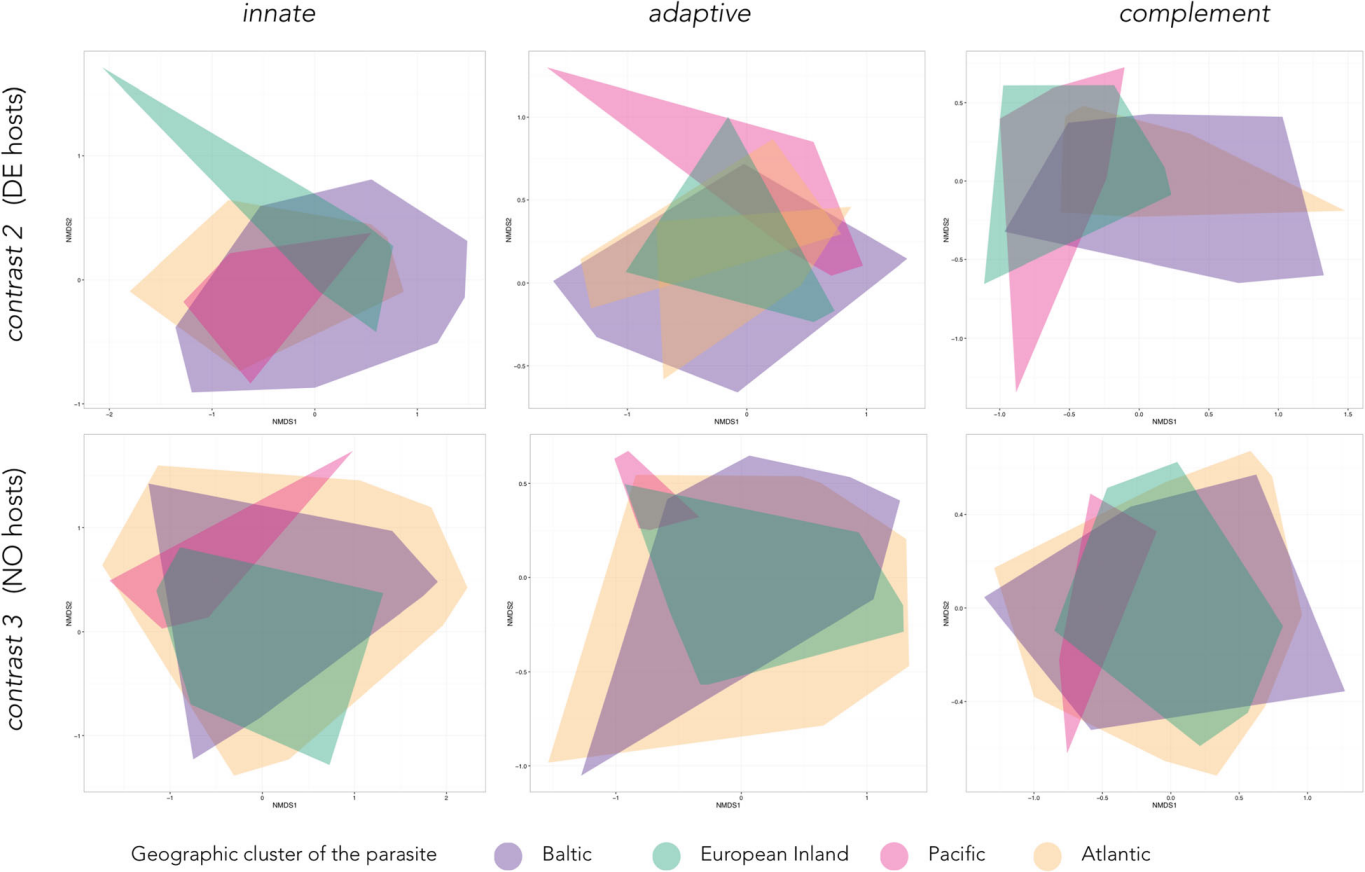
Infection with Pacific *S. solidus* drives significantly different multivariate gene expression patterns. Multivariate patterns in gene expression were visualized by non-metric multidimensional scaling (NMDS) on Euclidian distances and two dimensions using function metaMDS() from *vegan* [74]. Polygons were plotted using *ggplot2* [75]. NMDS were based on log10-transformed calibrated normalized relative quantities (CNRQ values) of twelve genes of innate immunity (*marco*, *mst1ra*, *mif*, *il-1β*, *tnfr1*, *saal1*, *tlr2, csf3r*, *p22*^*phox*^*, nkef-b, sla1, cd97*), nine genes of adaptive immunity (*stat4*, *stat6*, *igm*, *cd83*, *foxp3*, *tgf-β, il-16, mhcII, tcr-β*), or three genes of the complement system (*cfb, c7, c9*). Upper panel: data from DE hosts infected with seven different *S. solidus* strains from the four clustered localities (*contrast 2*); lower panel: data from NO hosts infected with nine different *S. solidus* strains from the four clustered localities (*contrast 3*). Color coding follows Fig. 1

Building on from the idea that *S. solidus* growth follows a geographic pattern, we asked whether the molecular phenotypes would show the same clustering. We studied the influence of *S. solidus* strain on stickleback immune gene expression by running pairwise PERMANOVAs within host populations (*contrast 2* or *contrast 3*) and tested (i) if gene expression differed within and/or between geographic clusters (Atlantic, Baltic, European Inland, Pacific) and (ii) if immune gene expression differed between sham-exposed controls and *S. solidus* infected sticklebacks for each parasite origin. Gene expression neither differed significantly within the clustered localities, nor between Baltic and Atlantic or European parasites, although the parasite indices differed considerably (Figs. 3 and 5). Immune gene expression profiles only differed between clustered localities if sticklebacks were infected with *S. solidus* from the Pacific (ECH) versus the Baltic or Atlantic region (Fig. 5).

In DE sticklebacks (*contrast 2*), Pacific *S. solidus* infection was associated with higher expression of innate immune genes (PERMANOVA_*innate*_: F_1,33_ = 3.88, *p* = 0.018), adaptive immune genes (PERMANOVA_*adaptive*_: F_1,33_ = 4.16, *p* = 0.013) and complement components (PERMANOVA_*complement*_: F_1,33_ = 8.1, *p* = 0.001) compared to infection with Baltic *S. solidus* (Table S13). Compared to infection with Atlantic *S. solidus*, Pacific *S. solidus* infection was associated with higher expression of adaptive immune genes (PERMANOVA_*adaptive*_: F_1,26_ = 5.84, *p* < 0.001) and complement components (PERMANOVA_*complement*_: F_1,26_ = 3.66, *p* = 0.016) in DE sticklebacks; only *mhcII* RNA levels were lower in Pacific *S. solidus* infections (F_1,26_ = 15.71, *p* = 0.0007; Additional file 1: Table S14).

In *contrast 3*, NO sticklebacks infected with Pacific *S. solidus* showed differential expression of genes of innate (PERMANOVA_*innate*_: F_1,29_ = 3.26, *p* = 0.006) and adaptive immunity (PERMANOVA_*adaptive*_: F_1,29_ = 5.8, *p* = 0.002) in comparison to infection with Baltic *S. solidus*. Seven innate immune genes (*marco*, *mif1*, *tnfr1*, *p22*^*phox*^, *nkef-b*, *sla1*, *cd97*) and five adaptive immune genes (*stat4*, *cd83*, *foxp3*, *tgf-β*, *il16*) were significantly higher expressed in Pacific *S. solidus* infections; only RNA levels of *mhcII* were significantly lower (Additional file 1: Table S15). In comparison to infection with Atlantic *S. solidus*, Pacific *S. solidus* infection was linked to higher expression of innate immune genes (PERMANOVA_*innate*_: F_1,47_ = 2.95, *p* = 0.014*)*, adaptive immune genes (PERMANOVA_*adaptive*_: F_1,47_ = 5.27, *p* = 0.004) and complement components (PERMANOVA_*complement*_: F_1,47_ = 5.16, *p* = 0.008) in NO hosts. Seven genes of innate immunity (*mst1ra*, *il-1β*, *tnfr1*, *p22*^*phox*^, *nkef-b*, *sla1*, *cd97*), seven genes of adaptive immunity (*stat4*, *igm*, *cd83*, *foxp3*, *tgf- β*, *il16*, *mhcII*) and complement *c9* were significantly higher expressed in NO sticklebacks infected with Pacific *S. solidus* in comparison to infection with Atlantic *S. solidus* (Additional file 1: Table S16).

We next tested if immune gene expression patterns differed between infected and control fish within *contrast 2* or *contrast 3*. Again, gene expression patterns were not related to parasite indices or size but strain-specific.

In DE hosts (*contrast 2*), expression of genes of all three functional arms of the stickleback’s immune system differed significantly between sham-exposed controls and fish infected with Pacific *S. solidus* (PERMANOVA_*innate*_: F_1,32_ = 7.51, *p* < 0.0001; PERMANOVA_*adaptive*_: F_1,32_ = 6.47, *p* < 0.001; PERMANOVA_*complement*_: F_1,32_ = 5.57, *p* = 0.007; Additional file 1: Table S17) or Scottish (NU) *S. solidus* (PERMANOVA_*innate*_: F_1,35_ = 4.89*, p* = 0.003; PERMANOVA_*adaptive*_: F_1,35_ = 3.925, *p* = 0.009; PERMANOVA_*complement*_: F_1,35_ = 4.75*, p* = 0.014; Table S18). Infection with Norwegian (SKO) *S. solidus* altered expression of adaptive immune genes (PERMANOVA_*adaptive*_: F_1,35_ = 8.76, *p* < 0.0001) and complement genes (PERMANOVA_*complement*_: F_1,35_ = 3.42, *p* = 0.028; Additional file 1: Table S19).

In NO sticklebacks (*contrast 3*), innate immune genes and complement components were differentially expressed between controls and hosts infected with Pacific *S. solidus* (PERMANOVA_*innate*_: F_1,26_ = 5.43*, p* = 0.0118; PERMANOVA_*complement*_: F_1,26_ = 7.61*, p* = 0.008; Additional file 1: Table S20). Adaptive immune genes were differentially expressed between controls and Atlantic (NU) *S. solidus* infections (PERMANOVA_*adaptive*_: F_1,39_ = 5.71, *p* = 0.002; Additional file 1: Table S20).

## Discussion

Parasites are important components of the host’s environment and a crucial agent of natural selection [5, 7, 8, 34, 35, 41]. The co-evolution between hosts and parasites entails complex dynamics, influencing host defense and parasite infectivity and virulence. We used controlled infection experiments of three-spined sticklebacks from two contrasting populations with a variety of *Schistocephalus solidus* strains in order to characterize specificity and consequences of divergent co-evolution in a vertebrate host-parasite association. We propose that main effects of the host and the parasite determine *S. solidus* virulence, whereas the interaction might play a minor role.

### Immunological differences between host populations

NO sticklebacks come from a population with high *S. solidus* prevalence and low parasite diversity [24, 36]. Since immune defence is costly and co-evolves with parasite virulence [7, 37–40], we hypothesized that NO sticklebacks evolved specific resistance against *S. solidus*. Infection rates did not differ significantly between host populations, but *S. solidus* plerocercoids were consistently smaller in NO hosts. This supports our hypothesis that NO hosts evolved increased resistance against *S. solidus* as inferred from parasite growth suppression [11, 41]. We found that controls from the DE population had higher immunological activity than NO controls (Fig. 4; SI.3–4). This is in line with the natural situation, as DE hosts are constantly challenged through high parasite diversity and abundance. However, the differences in immunological activation between the two host populations mostly converged upon infection: while immune gene expression profiles and respiratory burst activity of head kidney leukocytes differed significantly between controls, those parameters converged when fish were infected with *S. solidus* from most origins (Fig. 4; Additional file 1: Figure S1). This resembles the results from among-lake reciprocal transplant experiments [46] and comparisons of wild and laboratory-raised fish [47]. Consistently, these findings emphasize the importance of environmental effects on immune gene expression relative to genetic adaptation. We infer from our data that phenotypic plasticity in response to parasite infection is a stronger contributor to immunological activation than host genotype.

### Parasite strain specific immune gene expression

Host immune gene expression did not depend on *S. solidus* size or geographic cluster, but was parasite strain specific. Immune gene expression profiles differed between NO and DE controls and if fish were infected with Icelandic (ISC) *S. solidus* (*contrast 1*). Notably, Icelandic sticklebacks seem to be genetically distinct from other Atlantic populations [48].

Within DE hosts (*contrast 1*) and within NO hosts (*contrast 2*), expression profiles of infected fish did not differ between or within clustered parasite localities, but only if sticklebacks were infected with *S. solidus* from the Pacific (ECH) (Fig. 3). Those parasites originated from the geographically most distant population, indicating the potential of local adaptation at this scale [33]. Infection with Pacific *S. solidus* was consistently associated with high expression of most immune genes but low expression levels of *mhcII*. Major histocompatibility complex (MHC) class II molecules are important components of adaptive immunity and activate T-cell mediated humoral immune responses [49]. In our experiments, low expression of *mhcII* was often associated with low expression of the gene of T-cell receptor subunit TCR-β that is involved in MHC ligand binding (Additional file 1: SI.4). If a speculative active down-regulation of this arm of the immune system in allopatric combinations results from a direct manipulation by *S. solidus* remains to be answered.

In comparison to sham-exposed controls, Pacific *S. solidus* infection caused high expression of pro-inflammatory and complement genes in hosts of both populations (Additional file 1: Tables S17 and S20). Genes of adaptive immunity were highly expressed (*foxp3*) or down-regulated (*tcr-β* and *mhcII*) in DE hosts. A simultaneous up-regulation of *foxp3* is indicative of a T regulatory response [47] that potentially protects the host but may also enable parasite growth through anti-inflammatory activities. Indeed, ECH *S. solidus* were three times bigger in DE sticklebacks (Fig. 2). Pacific and Atlantic *S. solidus* reached similar sizes in DE hosts but, except for a potential involvement of *tcr-β* and/or *mhcII*, distinct genes were differentially expressed between hosts infected with parasites from different populations (Additional file 1: SI.4.3). We infer that (i) the relative parasite size and immune gene expression profiles are similar in infected fish of the two populations (similar parasite effect) but that (ii) complex ecological and co-evolutionary adaptations at different localities caused distinct levels of virulence and resistance.

### Geographic pattern of virulence

Parasite indices were strikingly similar between the two host populations with regard to the geographic origin of the parasite. *S. solidus* from Atlantic populations grew consistently larger and Baltic parasites were the smallest in both host types (Fig. 3). The geographic pattern of virulence in both host types highlights the parasite main effect. The greatest difference was the suppression of Pacific *S. solidus* growth through Atlantic (NO) sticklebacks relative to Baltic (DE) sticklebacks. Sticklebacks from the Atlantic region likely originate from the Pacific [50], so we suggest a relatively similar genetic background of Pacific and Atlantic *G. aculeatus – S. solidus* species pairs. Such a similarity could explain the higher resistance of Atlantic hosts against Pacific parasites. Baltic stickleback populations, in contrast, form a cluster that is distinct from European Inland populations [48]. This, again, is a pattern that we also see in *S. solidus* growth (Fig. 2). Thus, the geographic pattern of virulence corresponds to the host’s recolonization history after the last glaciation [48]. Based on these data and a previous study [35], we hypothesize that the parasite’s phylogeny resembles the phylogeny of its highly specific host. A genetic basis could explain the same clusters of *S. solidus* growth in both host types. Latitude or geographical distance between host and parasite source populations did not explain parasite size. This renders the question of what could have selected for different *S. solidus* types.

We propose that *S. solidus* evolved different life-history strategies in response to distinct selection by their hosts and habitat-specific trade-offs. Baltic *S. solidus* from NST, where *S. solidus* prevalence is extremely low [32], did not reach the proposed minimum weight (50 mg) for sexual reproduction in final hosts [28, 51, 52]. Baltic *S. solidus* from Swedish populations (OBB, GOT), where *S. solidus* prevalence is actually high (T. Henrich; pers. comm.), showed the same growth pattern. Hence, parasite prevalence might be one explanation [32, 34, 35, 41], but is certainly not the only cause for different growth strategies, especially in the light of ecological effects on exposure risk [33]. Another possible inference is that *S. solidus* from the Baltic region reach sexual competence at lower weights than those from other populations, which is supported by the fact that smaller worms can reproduce [26]. Nevertheless, mapping variation on fitness differences in the natural habitat remains to be investigated.

## Conclusions

We tested the specificity and immunological activation of three-spined sticklebacks *Gasterosteus aculeatus* towards various strains of the cestode *Schistocephalus solidus* at different stages of the infection process. (i) *S. solidus* infection rates were consistent among the two host populations whereas (ii) the growth of the parasite differed significantly among host populations and among parasite strains from different geographic clusters. Parasite indices were determined by main effects of the host and the parasite with nonsignificant interaction effects. (iii) Immune gene expression profiles were host-parasite combination specific, suggesting stronger interaction effects at this level of the infection process. Our results highlight the differences between mechanisms of distinct stages of the infection process and provide new insights into cestode growth suppression as a form of resistance [41].

We found constitutive immunological population differences but similar responses to infection. Our data provide evidence for (co-)evolutionary and ecological effects on immune functions that favour immunological heterogeneity. We propose that sticklebacks and *S. solidus* from a population with high *S. solidus* prevalence (NO) co-evolved high virulence and high resistance. The high resistance of NO hosts against *S. solidus* (host main effect) was not strain specific on an intermediate geographic scale (across Western Europe). On a larger geographic scale, parasites from the most distant (Pacific) population triggered elevated immunological parameters. The analogous clustering of parasite growth according to geography in the two host populations highlights the strong contribution of the parasite main effect on infection phenotypes. We suggest that patterns of local adaptation are either weak, absent or might be found at large scales [32–35].

## Methods

### Experimental hosts and parasites

Hosts and parasites were laboratory-raised first generation offspring from wild-caught individuals. Sticklebacks originated from lake Großer Plöner See, Germany (DE), and lake Skogseidvatnet, Norway (NO) and were kept in the institute’s aquaria system at 18 °C and a light:dark rhythm of 16:8 h. All fish were approximately nine months old at the start of the respective experiment. Sticklebacks were experimentally infected in 18 different combinations. We ran two experiments with essentially the same procedures. Each experiment was composed of three rounds using distinct fish families and parasite sibships. ‘Fish family’ refers to offspring from one pair of sticklebacks; ‘parasite sibship’ refers to offspring from one pair of worms. Parasite sibships from one origin are here referred to as ‘strain’. Sham-exposed controls were included in each round. A total of 1345 fish were analysed (Table 1; Table S1). We tested for host, parasite and host-parasite interaction effects using *contrast 1*. The respective infection experiments were run simultaneously and involved the exact same parasite sibships for both host populations, which should reduce any confounding factors. Parasite effects were further tested within each host type by using *S. solidus* strains from across the Northern Hemisphere (Table 1; Figs. 1 and 2). *Schistocephalus solidus* plerocercoids had been sampled from naturally infected sticklebacks from nine different locations (Fig. 2; Additional file 1: Table S1). The sampling sites cover four geographic areas corresponding to *G. aculeatus* phylogeny: the Atlantic region (NU, ISC, SKO), the Baltic region (OBB, NST, GOT), European Inland (SP, IBB), and the Pacific (ECH). The parasites were bred in vitro in the laboratory in 2012–2014. The eggs were kept at 4 °C in the dark.

### Infection experiments

*S. solidus* eggs developed at 20 °C for three weeks. A 3:8 h light:dark cycle and another light stimulus initiated hatching of the first larval stage (coracidia). Single coracidia were immediately fed to *Macrocyclops albidus* copepods (first intermediate hosts) from laboratory cultures. Copepods were kept at 16:8 h light:dark cycles at 18 °C and fed with *Paramecium* three times a week. Infection success was determined by inspection for procercoids (second larval stage) in vivo 7 to 11 DPE. On day 16, sticklebacks were exposed to single infected copepods or uninfected controls. By this time, *S. solidus* is infective to its second intermediate host and differences in infection success are unlikely to be caused by variation in ontogeny [53, 54]. The fish were starved for two days and isolated in individual tanks. We assigned numbers to each treatment group, i.e. worm sibship and the control, and used a random design for the exposure to avoid any observer bias. The fish were transferred to 16 L aquaria according to their numbers 24 h after exposure. The water was sieved in order to determine the number of ingested copepods per treatment. Sticklebacks were kept in aerated aquaria connected to a flow-through freshwater system at 18 °C and a light:dark rhythm of 16:8 h. The density of 20 individuals per aquarium was maintained by replacing dead fish with spine-clipped sticklebacks from the same fish family.

The fish were fed with frozen *Chironomidae* larvae three times a week but starved for two to four days before dissection. We dissected the fish in the laboratory 55 (+/− 2) DPE. Fish of every treatment group per experiment were dissected on each day. Sticklebacks were euthanized with MS222 (1 g/L), weighed and measured (standard length, i.e. without tail fin). The head kidneys, spleen, liver, gonads, and, if present, worms were weighted to the nearest 0.1 mg. The carcasses were stored on ice upon dissection. Head kidney cells were immediately prepared for flow cytometric analyses. Spleen, liver and worms were transferred to RNAlater*®* (Sigma R0901; tenfold volume per weight), kept at 4 °C for one day and stored at − 20 °C until further use.

### Phenotypic parameters

Infection rates were calculated with respect to the number of copepods that had not been ingested and include data from double infected hosts and fish that died before the day of dissection. The parasite index (PI) is a proxy for parasite size and host exploitation [32] and is calculated as the proportion of the total weight of an infected fish accounted for by the parasite [27]. The condition factor [42] and the hepatosomatic index (HSI) [43] are estimates of host condition. The splenosomatic index (SSI) [55] and head kidney index (HKI) were used as first proxies of immunological activation. The head kidney is the major immune organ in bony fish [44]. Thus, head kidney leukocytes (HKL) were studied in more detail [56] (Additional file 1: SI.3). Briefly, total cell numbers were determined by a modified protocol [57] of the Standard cell dilution assay [58]. Granulocytes and leukocytes were identified according to their FSC/SSC profiles using a Becton Dickinson FACS Calibur and BD CellQuest™ pro software (Version 6.0). We calculated a granulocyte to lymphocyte ratio (G/L ratio) as a rough activity estimate of the innate versus the adaptive immune system [59], and used a lucigenin-enhanced chemiluminescence assay [59, 60] to measure the phagocytic capacity of HKL by quantifying the respiratory burst reaction in relative luminescence units (RLUs). More details can be found in Additional file 1: SI.3.

### Gene expression analyses

Differential gene expression of *S. solidus* infected fish and sham-exposed controls was studied by quantitative real time reverse transcription PCR (RT-qPCR). Total RNA from spleen was extracted with the NucleoSpin®96 Kit (Macherey-Nagel) according to the manufacturer’s manual. Samples were thawed at 4 °C, transferred to new tubes, supplied with ß-mercaptoethanol (1% v/v) containing lysis buffer and homogenized for 2 × 3 min at 30 Hz using Tissue Lyser II (Qiagen). A DNase digestion step was included. RNA was eluted with 40 μL RNase-free H_2_O. RNA concentration and quality were measured spectrophotometrically (NanoDrop; Thermo Scientific). Samples with concentrations below 6 ng/μL or A_260_/A_280_ ratios < 1.9 were excluded. Reverse transcription was performed on 6.4 ng of total RNA using the Omniscript® RT Kit (Qiagen) with oligo dT priming and RNase inhibition (0.2 μL per reaction) at 37 °C for 60 min. 12.8 μL of sample RNA were used if the concentration was below 39 ng/μL. The cDNA was stored at − 20 °C and diluted 1:5 with RNase-free H_2_O before pre-amplification. Pre-amplification was performed with TaqMan® PreAmp Master Mix (Applied Biosystems) according to the manufacturer’s instructions with 14 cycles. The PCR product was diluted 1:5 with low TE buffer. Differences in transcription levels were tested using 96.96 Dynamic Array IFCs on a Biomark™ HD system (Fluidigm) according to the manufacturer’s protocol. EvaGreen was used as DNA binding dye. Samples were spread across four IFCs. All targets for a given sample were included in the same run and measured in triplicates (technical replicates). Inter-run calibrators, dilution series, and negative controls were included on each IFC. *Fluidigm Analysis software* was used to assess melting curves of all qPCR assays in order to confirm specific amplification. Samples with suspicious T_m_ profiles in more than two targets or failed amplifications were excluded. *Qbase + 3.0* (Biogazelle) was used for calculation of calibrated normalized relative quantities (CNRQ values). Replicates with variability (difference in quantification cycle, Cq) > 0.5 and wells with Cq > 28 were excluded, resulting in 94% pass rate. The average Cq was calculated as arithmetic mean; targets were scaled to average. We determined target and run specific amplification efficiencies. Expression stability of putative reference targets was inferred from geNorm M and Coefficient of Variation (CV) values [61, 62]. The most stably expressed reference targets *rpl13* and *ubc* (M = 0.133, CV = 0.046) were used for normalization. CNRQs were log10 transformed for analysis. Three missing values from gene *csf3r* and one missing value from *tlr2* were replaced by the mean expression of the respective gene. We analysed gene expression data of a total of 284 individuals from 18 different combinations including controls.

### Genes targeted in expression analyses

We used 28 different primer pairs targeting mRNA from immune related genes and putative reference genes (*b2m*, *ef1a*, *rpl13a*, *ubc*; described in [63]). Targets of interest covered genes of innate immunity (*cd97*, *csf3r*, *il-1β*, *marco*, *mif*, *mst1ra*, *nkef-b*, *tnfr1*, *saal1*, *tlr2*, *p22*^*phox*^, *sla1*), adaptive immunity (*cd83*, *foxp3*, *igm*, *il-16*, *stat4*, *stat6*, *tgf-β*, *mhcII*, *tcr-β*) and the complement system (*cfb, c7, c9*). Primers are described in [46, 47, 64] and in Piecyk, Ritter & Kalbe (*in review*); the detailed information can be found in Additional file 2.

### Statistical analyses

Statistical analyses were performed with R v. 3.2.0; [65]. We used (generalized) mixed effects models (GLMMs) from *nlme* [66] and *lme4* [67] to include random terms and fixed effects according to the experimental design. Infection rates were analysed by using the number of infected individuals as proportional data in GLMMs with binomial error structure and logit link function. The interaction of host and parasite was included in *contrast 1* (Table 1). Genotypic variation was generally accounted for by including parasite sibship or ‘round’, i.e. worm sibship x fish family combination, as random term. Models for fish parameters included the sex of the fish as another random effect to account for sex-specific differences. Model selection was based on the Akaike information criterion (AIC) and log likelihood ratio tests. Whenever needed, we incorporated heteroscedasticity in the model fit by definition of the varIdent variance structure for factorial variables. R^2^ values of mixed effects models [68, 69] were calculated with function sem.model.fits() from *piecewiseSEM* [70]. Significantly different groups were identified with glht() post hoc tests from *multcomp* [71] using Tukey’s all-pair comparisons or user defined contrasts according to the respective hypothesis. Multiple testing was accounted for by false discovery rate (FDR) correction [72]. Gene expression data was derived from infected and control fish from each family. Differential immune gene expression was analysed between groups within contrasts by multivariate statistics on data of all 24 immune genes and, if significant, according to functional groups (*innate*, *adaptive*, *complement*). Non-parametric permutational multivariate analyses of variance (PERMANOVA [72]) were calculated on Euclidian distance matrices [73] using function adonis() from *vegan* [74]. For each test, a random subset of 10,000 permutations was used; permutations were constrained within ‘round’. The weight of the fish was included as covariate to account for size related effects. Post hoc pairwise comparisons were FDR-corrected [72]. If multivariate statistics indicated significant differences, we used linear mixed models (LMMs) to identify which genes were differentially expressed. Again, we accounted for unequal variances and used FDR correction due to multiple testing. In each case, the raw *p*-values are reported. Data was plotted using *ggplot2* [75]; colours for plots and figures were chosen from the ColorBrewer palette [76]. Multivariate patterns in gene expression were visualized by non-metric multidimensional scaling (NMDS) on Euclidian distances and two dimensions (function metaMDS()); the contribution of each gene was plotted by use of the envfit() function (both implemented in *vegan*). The *maps* package [77] was used to draw the map of the sampling sites.

## Additional files

Additional file 1: Supplementary information. Supplementary tables and figures for ‘Specificity of resistance and geographic patterns of virulence in a vertebrate host-parasite system’. (PDF 2743 kb)

Additional file 2: Primer information. The primer information is cited from Piecyk, Ritter & Kalbe (*in review*). (PDF 108 kb)

## Abbreviations

CF: condition factor
CNRQ: calibrated normalized relative quantities
DE, NO: sticklebacks from Lake Großer Plöner See (Germany), Lake Skogseidvatnet (Norway)
DPE: days post exposure
ECH, GOT, IBB, ISC, NST, NU, OBB, SKO, SP: parasite strains from Echo Lake (Canada), Gotland (Sweden), Ibbenbürener Aa (Germany), Myvatn (Iceland), Neustädter Binnenwasser (Germany), North Uist (Scotland), Obbola (Sweden), Lake Skogseidvatnet (Norway), Xinzo de Limia (Spain)
(G)LMM: (generalized) linear mixed model
FDR: false discovery rate
G/L: granulocyte to lymphocyte ratio
HKI: head kidney index
HKL: head kidney leukocytes
HSI: hepatosomatic index
IFC: integrated fluidic circuits
PERMANOVA: permutational multivariate analysis of variance
PI: parasite index
RLU: relative luminescence units
ROS: reactive oxygen species
SSI: splenosomatic index
