# Correction to: Specificity of resistance and geographic patterns of virulence in a vertebrate hostparasite system

**DOI:** 10.1186/s12862-019-1419-y

**Published:** 2019-05-13

**Authors:** Agnes Piecyk, Olivia Roth, Martin Kalbe

**Affiliations:** 1 Department of Evolutionary Ecology, Max Planck Institute for Evolutionary Biology, August-Thienemann-Straße 2, 24306 Plön, Germany; 2 Marine Evolutionary Ecology, GEOMAR Helmholtz Centre for Ocean Research Kiel, Düsternbrookerweg 20, 24105 Kiel, Germany

**Correction to: BMC Evol Biol**

**https://doi.org/10.1186/s12862-019-1406-3**

After publication of the original article [1], the authors have notified us that the incorrect version of Fig. 4 was used. Below you can find the both incorrect and correct versions of the figure.

**Fig. 4:**
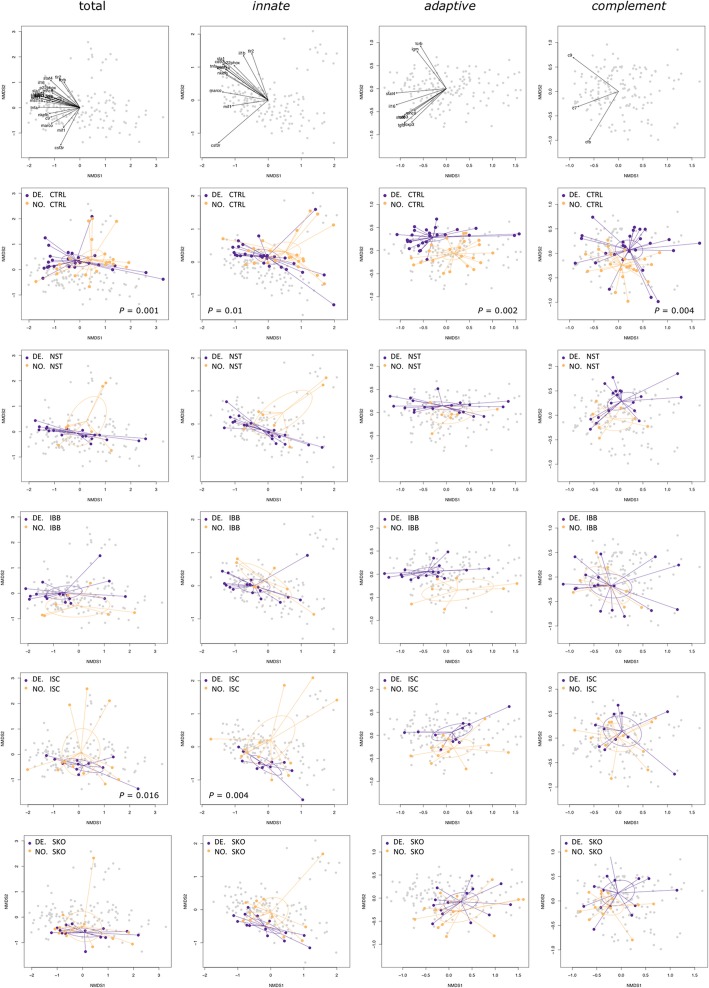
Multivariate gene expression patterns differ between DE and NO sticklebacks. Non-metric multidimensional scaling (NMDS) plots on Euclidian distances and two dimensions comparing data from NO and DE sticklebacks (*contrast 1*). NMDS were based on log10-transformed calibrated normalized relative quantities (CNRQ values) of all 24 immune genes, twelve genes of innate immunity (*marco*, *mst1ra*, *mif*, *il-1β*, *tnfr1*, *saal1*, *tlr2, csf3r*, *p22*^*phox*^*, nkef-b, sla1, cd97*), nine genes of adaptive immunity (*stat4*, *stat6*, *igm*, *cd83*, *foxp3*, *tgf-β, il-16, mhcII, tcr-β*), or three genes of the complement system (*cfb, c7, c9*). Each dot represents one individual; colors refer to the host population. Ellipses represent 95% confidence intervals. *P*-values are shown if significant after FDR-correction. The contribution of each gene is shown in the first row. The second row shows data from sham-exposed (CTRL) sticklebacks. The third to sixth row show data from infected individuals. Function metaMDS() was used to plot the NMDS; the contribution of each gene was plotted by use of the envfit() function (both functions are implemented in R package *vegan* [74])

**Fig. 4:**
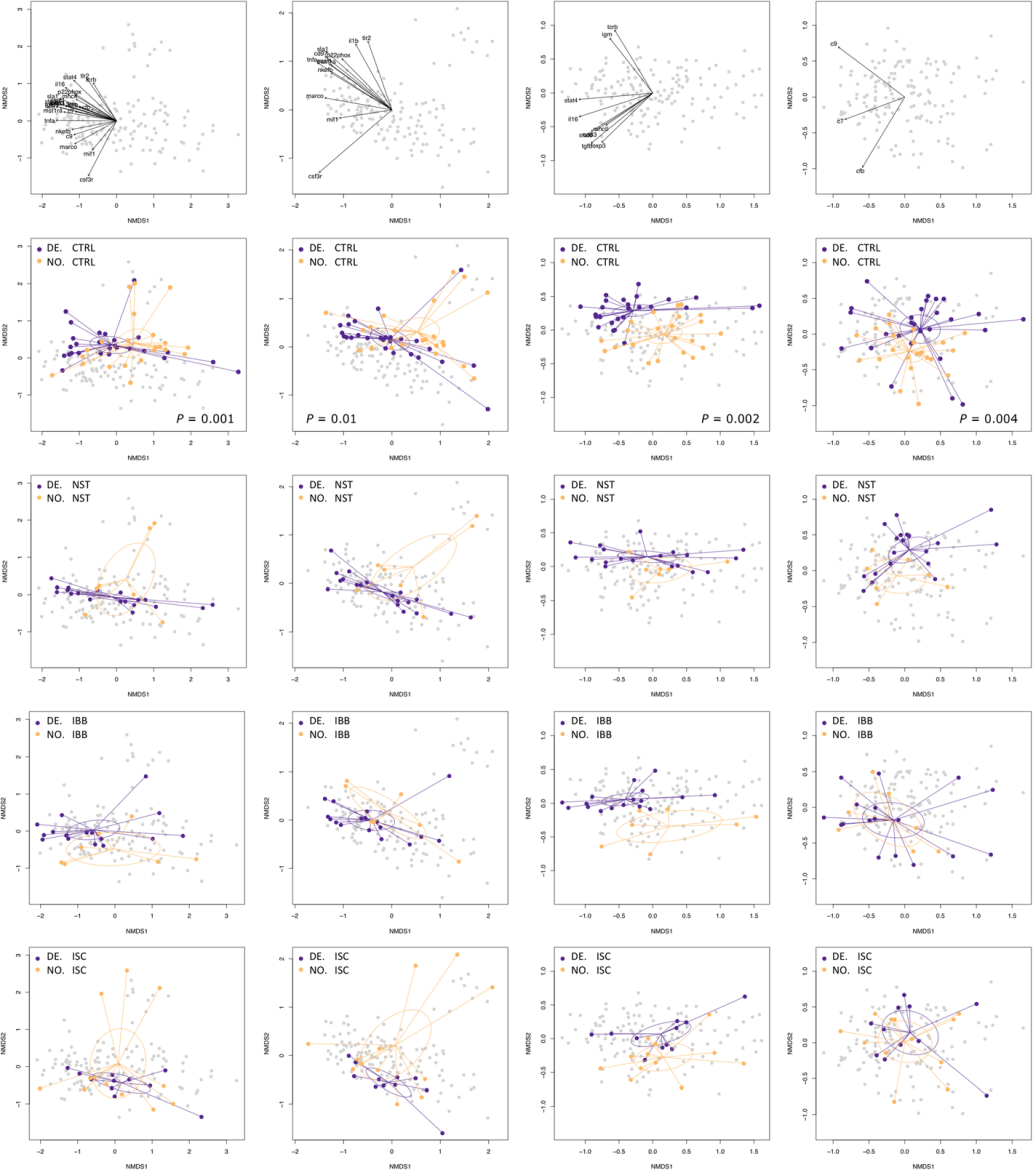
Multivariate gene expression patterns differ between DE and NO sticklebacks. Non-metric multidimensional scaling (NMDS) plots on Euclidian distances and two dimensions comparing data from NO and DE sticklebacks (*contrast 1*). NMDS were based on log10-transformed calibrated normalized relative quantities (CNRQ values) of all 24 immune genes, twelve genes of innate immunity (*marco*, *mst1ra*, *mif*, *il-1β*, *tnfr1*, *saal1*, *tlr2, csf3r*, *p22*^*phox*^*, nkef-b, sla1, cd97*), nine genes of adaptive immunity (*stat4*, *stat6*, *igm*, *cd83*, *foxp3*, *tgf-β, il-16, mhcII, tcr-β*), or three genes of the complement system (*cfb, c7, c9*). Each dot represents one individual; colors refer to the host population. Ellipses represent 95% confidence intervals. *P*-values are shown if significant after FDR-correction. The contribution of each gene is shown in the first row. The second row shows data from sham-exposed (CTRL) sticklebacks. The third to sixth row show data from infected individuals. Function metaMDS() was used to plot the NMDS; the contribution of each gene was plotted by use of the envfit() function (both functions are implemented in R package *vegan* [74])

The original article has been corrected.
