# Supplementary material for: Specificity of resistance and geographic patterns of virulence in a vertebrate host-parasite system
Source: BMC Evol Biol. 2019 Mar 19;19:80. doi: 10.1186/s12862-019-1406-3 (PMC6425677; doi:10.1186/s12862-019-1406-3)
Supplement: Supplementary file 1 — Supplementary information. Supplementary tables and figures for ‘Specificity of resistance and geographic patterns of virulence in a vertebrate host-parasite system’. (PDF 2743 kb) [file 12862_2019_1406_MOESM1_ESM.pdf]

Additional file 1: Supplementary information

SI.1 Supplementary information on infection rates 1

SI.2 Supplementary information on parasite indices (*contrast 1*) 5

SI.3 Supplementary information on host condition and immunological parameters 7

SI.4 Supplementary information on host immune gene expression (*contrast 1*) 13

SI.4.1 Stickleback immune gene expression differences between populations 13

SI.5 Supplementary information for *contrast 2* and *contrast 3* 16

SI.5.1 Stickleback immune gene expression according to clustered localities of parasites 18

SI.5.2 Stickleback immune gene expression in infected versus control fish 22

SI.5.3 NMDS: infected versus control DE sticklebacks (*contrast 2*) 27

SI.5.4 NMDS: infected versus control NO sticklebacks (*contrast 3*) 31

SI.5.5 NMDS: infected versus control DE sticklebacks (in *contrast 1*) 35

References 36

Table S1. Parasite sampling sites

ID	Origin		Geo-coordinates
ECH	Echo Lake, Vancouver Island	Canada	49°98’N, 125°41’W
ISC	Lake Myvatn	Iceland	65°39’N, 16°57’W
NU	North Uist (Loch Eubhal/ Grogary)	Scotland	57°34’N, 07°17’W
SKO	Skogseidvatnet	Norway	60°13’N, 05°53’E
SP	Xinzo de Limia	Spain	42°08’N, 07°39’W
IBB	Ibbenbürener Aa	Germany	52°17’N, 07°36’E
NST	Neustädter Binnenwasser	Germany	54°06’N, 10°48’E
GOT	Gotland	Sweden	57°54’N, 18°56’E
OBB	Obbola	Sweden	63°39’N, 20°17’E

SI.1 Supplementary information on infection rates

If not stated otherwise, infection rates were calculated by using the number of infected individuals as proportional data in generalized mixed effects models (GLMMs) with binomial error structure and logit link function using the glmer() function of the *lme4* R package (Bates et al., 2014). Significantly different groups were identified with glht() post hoc tests from the

multcomp package (Hothorn et al., 2008). Infection rates differed considerably between parasite sibships and fish families; some parasite sibships failed to infect any fish. According to our experimental design, however, we did not test for fish family or parasite sibship effects. Parasite sibship was included as a random factor in analyses of infection rates in copepods; the random term 'round' (i.e. parasite sibship x fish family combination) was included in all analyses of the interaction between *S. solidus* and its fish hosts.

As expected for the unspecific first intermediate host, *S. solidus* from every origin managed to infect *M. albidus* copepods. We tested for potential differences in infection rates in copepods between the two years of the experiment by using data from parasite sibships that were used in both years (Table S2). Indeed, infection rates of parasites from NU and SKO were significantly higher in 2014 (NU: z = 4.472, $p < 0.0001$; SKO: z = 6.214, $p < 0.0001$). Testing each year separately, infection rates did not differ significantly between parasite populations in 2014; in 2015, ISC *S. solidus* infected significantly more copepods than parasites from GOT (z = -5.289, $p < 0.001$), NU (z = 4.416, $p < 0.001$), OBB (z = 3.615, $p < 0.01$), SKO (z = 3.948, $p < 0.01$), SP (z = 4.115, $p < 0.01$); IBB *S. solidus* infected significantly more copepods than *S. solidus* from GOT (z = -4.638, $p < 0.001$), NU (z = 3.76, $p < 0.01$), SKO (z = 3.275, p = 0.029), and SP (z = 3.453, p = 0.016); ECH *S. solidus* infected significantly more copepods than *S. solidus* from GOT (z = 4.148, $p < 0.01$) and NU (z = 3.299, p = 0.027). Using the sibship of the parasite as explanatory instead of the origin improved the model fit, pointing towards sibship- rather than origin-effects. Interestingly, Pacific (ECH) parasites had the highest infection rates in copepods and the lowest infection rates in sticklebacks. However, overall, and consistent with previous publications (Hammerschmidt and Kurtz, 2005), infection rates in copepods did not influence infection rates in fish.

Infection rates in fish did not differ significantly between the two years of the experiment (DE data; $X^2_5 = 9.42$, p = 0.094). *S. solidus* origin influenced the infection rates in NO hosts (X^2_8 = 21.619, p = 0.006). This was driven by significant differences between infections with NU versus ECH parasites (z = -3.446, p = 0.016). NU *S. solidus* had the overall highest infection rate (average: 40 %) and ECH *S. solidus* had the lowest infection rate (average: 9 %). The variance terms for the random effect differed between the experiments, which indicates different parasite sibship x fish family effects; namely, lower variance in DE in *contrast 1*. Fish

from the naturally highly parasitized Norwegian (NO) population ate considerably less infected copepods than DE fish, so we tested for a possible link between the number of ingested copepods and infection success. There was no consistent pattern; the number of infected copepods correlated with an increase or decrease of the infection rates, dependent on the origin of the parasite and the fish population (not shown). Accordingly and in line with the literature (Wedekind and Milinski, 1996), our data does not indicate avoidance behaviour.

Table S2. Infection rates of *S. solidus* in its first intermediate host (*M. albidus*)

Year of the experiment	Parasite sibship	Parasite origin	Infected copepods	Uninfected copepods	Infection rate	Average per origin and experiment
2014	ECH_3x10	ECH	51	38	0.57	
2014	ECH_6x23	ECH	69	17	0.80	
2014	ECH_9x14	ECH	61	17	0.78	0.72
2014	GOT_10x12	GOT	44	42	0.51	
2014	GOT_13x8	GOT	24	62	0.28	
2014	GOT_1x5	GOT	48	38	0.56	0.45
2014	NST_13x14	NST	51	29	0.64	
2014	NST_2x7	NST	46	37	0.55	
2014	NST_8x9	NST	43	80	0.35	0.51
2014	NU_10x14	NU	60	28	0.68	
2014	NU_4x12	NU	21	65	0.24	
2014	NU_8x17	NU	56	21	0.73	0.55
2014	OBB_11x48	OBB	39	73	0.35	
2014	OBB_18x20	OBB	54	30	0.64	
2014	OBB_5x16	OBB	48	25	0.66	0.55
2014	SKO_18x49	SKO	45	36	0.56	
2014	SKO_18x57	SKO	52	33	0.61	
2014	SKO_57x58	SKO	49	36	0.58	0.58
2014	SP_10x12	SP	55	33	0.63	
2014	SP_14x19	SP	19	23	0.45	
2014	SP_1x13	SP	22	66	0.25	0.44
2015	ECH_3x10	ECH	49	24	0.67	
2015	ECH_6x23	ECH	60	28	0.68	
2015	ECH_9x14	ECH	53	32	0.62	0.66
2015	GOT_13x8	GOT	28	71	0.28	
2015	GOT_1x5	GOT	39	71	0.35	
2015	GOT_9x6	GOT	34	64	0.35	0.33
2015	IBB_35	IBB	76	56	0.58	
2015	IBB_39	IBB	117	55	0.68	
2015	IBB_41	IBB	123	35	0.78	0.68
2015	ISC_59	ISC	103	31	0.77	
2015	ISC_61	ISC	121	47	0.72	
2015	ISC_70	ISC	124	55	0.69	0.73
2015	NST_13x14	NST	76	66	0.54	
2015	NST_2x7	NST	86	65	0.57	
2015	NST_8x9	NST	92	80	0.53	0.55
2015	NU_10x14	NU	36	54	0.40	
2015	NU_5x18	NU	30	85	0.26	
2015	NU_8x17	NU	57	50	0.53	0.40
2015	OBB_11x48	OBB	44	50	0.47	
2015	OBB_18x20	OBB	33	54	0.38	
2015	OBB_5x16	OBB	46	43	0.52	0.45
2015	SKO_18x57	SKO	63	111	0.36	
2015	SKO_26x44	SKO	106	41	0.72	
2015	SKO_57x58	SKO	43	131	0.25	0.44
2015	SP_10x12	SP	55	75	0.42	
2015	SP_1x13	SP	44	44	0.50	
2015	SP_8x17	SP	43	82	0.34	0.42

Table S3. Infection rates and *S. solidus* size in DE and NO *G. aculeatus*

Year of the experiment	Fish family	Fish origin	Parasite sibship	Parasite origin	Total fish	Exposed fish	Infected fish	Uninfected fish	Infection rate	Mean weight	Mean PI
2014	GPS_16x6	DE	ECH_3x10	ECH	20	19	2	17	0.11	88.3	13.53
2014	GPS_16x6	DE	GOT_10x12	GOT	20	20	3	17	0.15	48.53	6.8
2014	GPS_16x6	DE	NST_8x9	NST	20	20	9	11	0.45	31.63	5.14
2014	GPS_16x6	DE	NU_8x17	NU	20	20	6	14	0.30	75.28	11.62
2014	GPS_16x6	DE	OBB_11x48	OBB	20	19	4	15	0.21	42.88	6.43
2014	GPS_16x6	DE	SKO_57x58	SKO	20	20	5	15	0.25	102.72	15.33
2014	GPS_16x6	DE	SP_14x19	SP	18	17	7	10	0.41	61.27	10.97
2014	GPS_24x29	DE	ECH_9x14	ECH	20	20	2	18	0.10	96.4	13.15
2014	GPS_24x29	DE	GOT_13x8	GOT	20	20	0	20	0.00	na	na
2014	GPS_24x29	DE	NST_13x14	NST	20	20	4	16	0.20	25.6	4.18
2014	GPS_24x29	DE	NU_10x14	NU	20	20	4	16	0.20	80.5	15.2
2014	GPS_24x29	DE	OBB_18x20	OBB	20	20	2	18	0.10	18.7	3.74
2014	GPS_24x29	DE	SKO_18x49	SKO	20	20	0	20	0.00	na	na
2014	GPS_24x29	DE	SP_10x12	SP	20	18	0	18	0.00	na	na
2014	GPS_5x3	DE	ECH_6x23	ECH	20	19	3	16	0.16	68.57	12.34
2014	GPS_5x3	DE	GOT_1x5	GOT	20	19	5	14	0.26	41.5	6.75
2014	GPS_5x3	DE	NST_2x7	NST	20	20	4	16	0.20	42.6	6.71
2014	GPS_5x3	DE	NU_4x12	NU	18	17	1	16	0.06	94.96	13.73
2014	GPS_5x3	DE	OBB_5x16	OBB	20	19	4	15	0.21	23.83	4.48
2014	GPS_5x3	DE	SKO_18x57	SKO	20	20	7	13	0.35	98.27	14.74
2014	GPS_5x3	DE	SP_1x13	SP	20	20	1	19	0.05	75	9.96
2015	GPS_117x111	DE	IBB_39	IBB	20	19	10	9	0.53	89.95	11.04
2015	GPS_117x111	DE	ISC_70	ISC	20	19	5	14	0.26	106.74	14.19
2015	GPS_117x111	DE	NST_8x9	NST	20	20	8	12	0.40	38.5	5.1
2015	GPS_117x111	DE	SKO_57x58	SKO	13	12	3	9	0.25	109.53	14.95
2015	GPS_125x105	DE	IBB_35	IBB	20	20	5	15	0.25	75.56	15.89
2015	GPS_125x105	DE	ISC_59	ISC	20	20	2	18	0.10	70.95	16.47
2015	GPS_125x105	DE	NST_2x7	NST	20	18	2	16	0.11	47.3	8.87
2015	GPS_125x105	DE	SKO_26x44	SKO	20	20	4	16	0.20	131.3	21.55
2015	GPS_22x4	DE	IBB_41	IBB	20	20	3	17	0.15	87.63	13.32
2015	GPS_22x4	DE	ISC_61	ISC	20	16	5	11	0.31	89.34	13.8
2015	GPS_22x4	DE	NST_13x14	NST	20	20	10	10	0.50	48.07	7.56
2015	GPS_22x4	DE	SKO_18x57	SKO	20	20	7	13	0.35	136.99	20.29
2015	SKO_10x6	NO	ECH_3x10	ECH	20	18	4	14	0.22	25.15	4.12
2015	SKO_10x6	NO	GOT_13x8	GOT	19	17	4	13	0.24	12.83	2.18
2015	SKO_10x6	NO	IBB_41	IBB	20	17	2	15	0.12	46.7	7.08
2015	SKO_10x6	NO	ISC_61	ISC	20	18	6	12	0.33	50.93	7.59
2015	SKO_10x6	NO	NST_13x14	NST	20	16	4	12	0.25	3.85	0.85
2015	SKO_10x6	NO	NU_5x18	NU	14	14	7	7	0.50	59.57	9.01
2015	SKO_10x6	NO	OBB_18x20	OBB	20	17	10	7	0.59	6.89	1.18
2015	SKO_10x6	NO	SKO_18x57	SKO	20	14	8	6	0.57	69.08	10.91
2015	SKO_10x6	NO	SP_8x17	SP	20	18	5	13	0.28	45.18	7.43
2015	SKO_11x2	NO	ECH_9x14	ECH	20	17	0	17	0.00	na	na
2015	SKO_11x2	NO	GOT_1x5	GOT	20	19	2	17	0.11	10.3	1.71
2015	SKO_11x2	NO	IBB_39	IBB	20	17	2	15	0.12	55.85	8.75
2015	SKO_11x2	NO	ISC_70	ISC	20	14	5	9	0.36	45.04	6.7
2015	SKO_11x2	NO	NST_8x9	NST	20	16	2	14	0.13	10.95	1.75
2015	SKO_11x2	NO	NU_8x17	NU	20	16	6	10	0.38	88.65	12.31
2015	SKO_11x2	NO	OBB_11x48	OBB	20	15	1	14	0.07	2.8	0.5
2015	SKO_11x2	NO	SKO_57x58	SKO	20	19	2	17	0.11	123	13.65
2015	SKO_11x2	NO	SP_10x12	SP	20	16	2	14	0.13	51.8	7.16
2015	SKO_4x17	NO	ECH_6x23	ECH	20	19	1	18	0.05	42.9	7.28
2015	SKO_4x17	NO	GOT_9x6	GOT	20	17	1	16	0.06	0.6	0.11
2015	SKO_4x17	NO	IBB_35	IBB	20	17	3	14	0.18	53.97	8.62
2015	SKO_4x17	NO	ISC_59	ISC	20	10	1	9	0.10	89.3	13.89
2015	SKO_4x17	NO	NST_2x7	NST	20	15	2	13	0.13	7.2	1.22
2015	SKO_4x17	NO	NU_10x14	NU	20	16	5	11	0.31	68.42	10.8
2015	SKO_4x17	NO	OBB_5x16	OBB	20	18	1	17	0.06	3.4	0.51
2015	SKO_4x17	NO	SKO_26x44	SKO	20	14	4	10	0.29	83.78	12.78
2015	SKO_4x17	NO	SP_1x13	SP	20	16	2	14	0.13	34.15	5.46

Table S4. Host and parasite effects and their interaction on infection rates

**Host effect, parasite effect and interaction**				
Data subset	Explanatory	Df	Chisq	p-value
Contrast 1 (simultaneously infected DE and NO hosts)	*S. solidus* origin	3	0.8817	0.82985
	Host population	1	2.2658	0.13226
	S. solidus origin : host population	3	6.4206	0.09285
**Parasite effect**				
Data subset	Explanatory	Df	Chisq	p-value
Contrast 2 (DE in 2014)	*S. solidus* origin	6	7.1518	0.307
Contrast 3 (NO in 2015)	*S. solidus* origin	8	21.619	0.00567

**Differences between the two years of the experiment**				
Data subset	Explanatory	Df	Chisq	p-value
DE hosts infected in 2014 and 2015	*S. solidus* origin	1	1.1665	0.28013
	Round	5	9.416	0.09358
	S. solidus origin : round	3	3.4414	0.32844

Sticklebacks from two different host populations (DE and NO) were exposed to *S. solidus* parasites from nine different locations in three experiments over two consecutive years (2014 and 2015). The infection rates were analyzed as proportional data (accounting for the copepods that were not ingested) with binomial error structure. We tested for differences between the years by using data of hosts that were exposed to the same sibships in the two years of the experiment. The respective generalized linear model (GLM) included 'round' (fish family x parasite sibship combination) and the interaction with *S. solidus* origin as an explanatory. Host and parasite effects were analyzed with GLMMs including 'round' as random effect.

SI.2 Supplementary information on parasite indices (*contrast 1*)

Table S5. The effect of host and parasite population on parasite indices

Explanatory	numDF	denDF	F-value	p-value	R^2
Host population	1	95	23.48201	< 0.0001	0.8934325
S. solidus origin	3	95	78.93636	< 0.0001	
Host population : *S. solidus* origin	3	95	0.99526	0.3986	

Sticklebacks were infected with single *S. solidus* parasites from NST, IBB, ISC, or SKO. The linear mixed model (LMM) included 'round', i.e. host and parasite genotype combinations, as random intercept. The R^2 includes the effect of the random term and was calculated according to (Nakagawa and Schielzeth, 2013; Johnson, 2014; Lefcheck, 2016).

Table S6. Post hoc testing using manually defined contrast to determine differences between fish populations.

	Parasite origin	Estimate	Std. Err	t-value	Pr(>|t|)
DE vs NO fish	IBB	-5.1459	1.0619	-4.846	< 0.0001
	ISC	-6.5330	1.0289	-6.350	< 0.0001
	NST	-5.9784	0.7431	-8.045	< 0.0001
	SKO	-7.7660	1.1629	-6.678	< 0.0001

Table S7. Post hoc testing using manually defined contrast to determine differences between origin of the parasites.

	Parasite origins			Estimate	Std. Error	t-value	Pr(>|t|)
DE fish	ISC	vs	IBB	1.6325	0.9230	1.769	0.4925
	NST	vs	IBB	-5.9838	0.7002	-8.545	<0.001
	SKO	vs	IBB	6.2738	1.0076	6.227	<0.001
	NST	vs	ISC	-7.6163	0.8279	-9.200	<0.001
	SKO	vs	ISC	4.6413	1.1015	4.214	<0.001
	SKO	vs	NST	12.2576	0.9186	13.344	<0.001
NO fish	ISC	vs	IBB	0.2453	1.1679	0.210	1.0000
	NST	vs	IBB	-6.8163	1.0961	-6.219	<0.001
	SKO	vs	IBB	3.6537	1.2217	2.991	0.0353
	NST	vs	ISC	-7.0617	0.9627	-7.335	<0.001
	SKO	vs	ISC	3.4083	1.1063	3.081	0.0273
	SKO	vs	NST	10.4700	1.0327	10.139	<0.001

SI.3 Supplementary information on host condition and immunological parameters

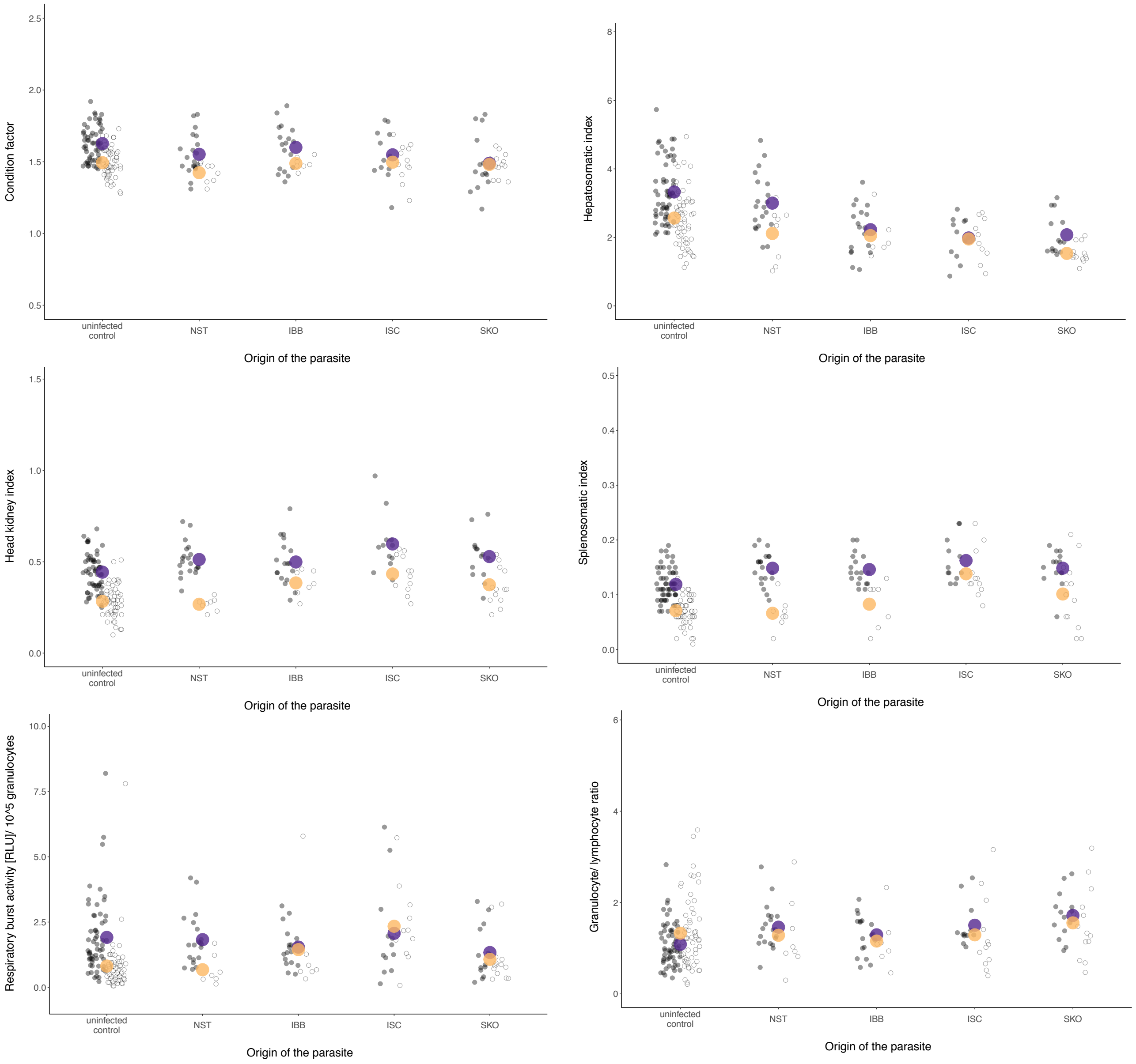

Figure S1. Phenotypic differences between NO (orange) and DE (violet) sticklebacks (*contrast 1*). The fish were either sham-exposed or infected with single *S. solidus* parasites from the Baltic (NST: Neustädter Binnenwasser, Germany), the European Inland (IBB: Ibbenbürener Aa, Germany), or the Atlantic region (ISC: Lake Myvatn, Iceland; SKO: Lake Skogseidvatnet, Norway). The parasite sibships, i.e. genotypes, were the same for both host populations. The fish were dissected 55 (+/- 2) DPE.

We determined the overall condition (condition factor, CF, the ratio between the observed weight W (in g) and the expected weight at a given length L (in cm): CF = 100 * W/L^b. The expected weight depends on the exponent b, which is characteristic for each fish population and was calculated by regression analysis of logarithm-transformed data of the length and the weight of all fish from each experiment, (Frischknecht, 1993)) and estimates of metabolic reserves (hepatosomatic index, HSI = 100 * W_L/ W, with W_L representing the weight of the liver, (Chellappa et al., 1995)) and immunological activity (splenosomatic index, SSI = 100 * W_S/ W, with W_s representing the weight of the spleen, (Seppänen et al., 2009); head kidney index, HKI, the weight of the head kidney in relation to body weight). Numbers of granulocytes and lymphocytes in 0.5 mL head kidney leukocyte (HKL) cell suspensions were used to calculate the granulocyte to lymphocyte (G/L) ratio as a proxy for the activity of the innate versus the adaptive immune system. Relative light units (RLUs) in a lucigenin-enhanced chemiluminescence assay quantify the production of reactive oxygen species (ROS) and hence phagocytic capacity of HKL.

Cell suspensions of HKL were prepared by forcing tissue samples through a 40 µm nylon mesh (BD Falcon, USA). The cells were transferred to a 96 deep well plate and rinsed twice in R-90 (90% (v/v) RPMI 1640 in distilled water) at 600 g for 10 min at 4 °C. Total cell numbers were determined by a modified protocol (Scharsack et al., 2004) of the Standard cell dilution assay (Pechhold et al., 1994). Therefore, each sample was supplemented with 2 mg/L propidium iodide (Sigma Aldrich) and 3 x 10^4 green fluorescent reference particles (4 µm, Polyscience, USA). FSC/SSC characteristics were measured in linear mode for one minute or for up to 10,000 events using a Becton Dickinson FACS Calibur and BD CellQuest™ pro software (Version 6.0). Propidium iodide positive (i.e. dead) cells and cellular debris (low FSC characteristics) were excluded from further analyses. Granulocytes and leukocytes were identified according to their FSC/SSC profiles. The numbers of viable granulocytes and lymphocytes in 0.5 mL were used to calculate the granulocyte to lymphocyte ratio (G/L ratio) (Kurtz et al., 2004). A lucigenin-enhanced chemiluminescence (CL) assay (Scott and Klesius, 1981; Kurtz et al., 2004) was used for functional analysis of innate immune activity. The CL assay measures the phagocytic capacity of HKL by quantifying the respiratory burst reaction in relative luminescence units (RLUs). Briefly, 10^5 live cells per sample were supplemented with

50 µg lucigenin (Sigma M 8010) and incubated at 18 °C and 2% CO_2 for 30 min. Zymosan (Sigma Z 4250) was added at a final concentration of 0.75 µg/µL to stimulate the production of reactive oxygen species (ROS). Chemiluminescence was measured every 3 min for 3.5 hours (Berthold Technologies luminometer) and the area under the kinetic curve (calculated with Win Glow 2000 professional software) was used for analyses. The RLU was standardized by division by the mean RLU of the negative controls (wells containing buffer without head kidney cells) for each day and by division by the number of vital granulocytes of the respective sample. Unfortunately, we could not obtain enough cells from every fish (data was missing from 13 samples) and thus analyzed production of reactive oxygen species of a total of 1430 different samples. Controls (medium without cells) were missing for one round in 2015. Values for those controls were inferred from data from empty wells in relation to controls.

Testing these condition and immunity related indices in each experiment, DE sticklebacks (*contrast 2*; Figure S2) showed significantly elevated immune parameters if they were infected with Pacific *S. solidus*: the head kidneys were larger (LMM; $p < 0.001$), the G/L ratio was significantly higher in comparison to all but SKO-infected fish (LMM; $p < 0.001$) and the head kidney's potential to produce reactive oxygen species was higher in comparison to controls (LMM; $p < 0.001$) and SKO-parasite infected fish (LMM; $p = 0.005$). The fish had significantly lower body condition than their respective controls if infected with Spanish parasites (LMM, $p < 0.001$) (Figure S2). In *contrast 1*, DE fish had significantly lower body condition than respective controls if infected with SKO-parasites (LMM; $p = 0.003$). The Hepatosomatic index was significantly smaller when fish were infected with fast growing *S. solidus* from IBB, ISC or SKO (LMMs; $p < 0.001$). Compared to controls, spleens were enlarged if fish were infected with parasites from ISC (LMM; $p < 0.001$), NST (LMM; $p < 0.001$) or SKO (LMM; $p = 0.003$). Head kidneys were larger in ISC-infected fish than in control fish (LMM; $p < 0.001$) and the G/L ratio was significantly higher in SKO-infected fish than in control fish (LMM; $p < 0.001$) (Figure S3). Relative to the control, NO sticklebacks had significantly lower Hepatosomatic indices when they were infected with sympatric (SKO-) *S. solidus* parasites (LMM; $p < 0.001$). The Splenosomatic index was higher in ISC-parasite infected fish in comparison to controls and NST-parasite infected fish (LMMs; each $p < 0.001$). Head kidney related immune parameters did not differ between infected and uninfected NO sticklebacks (Figure S4).

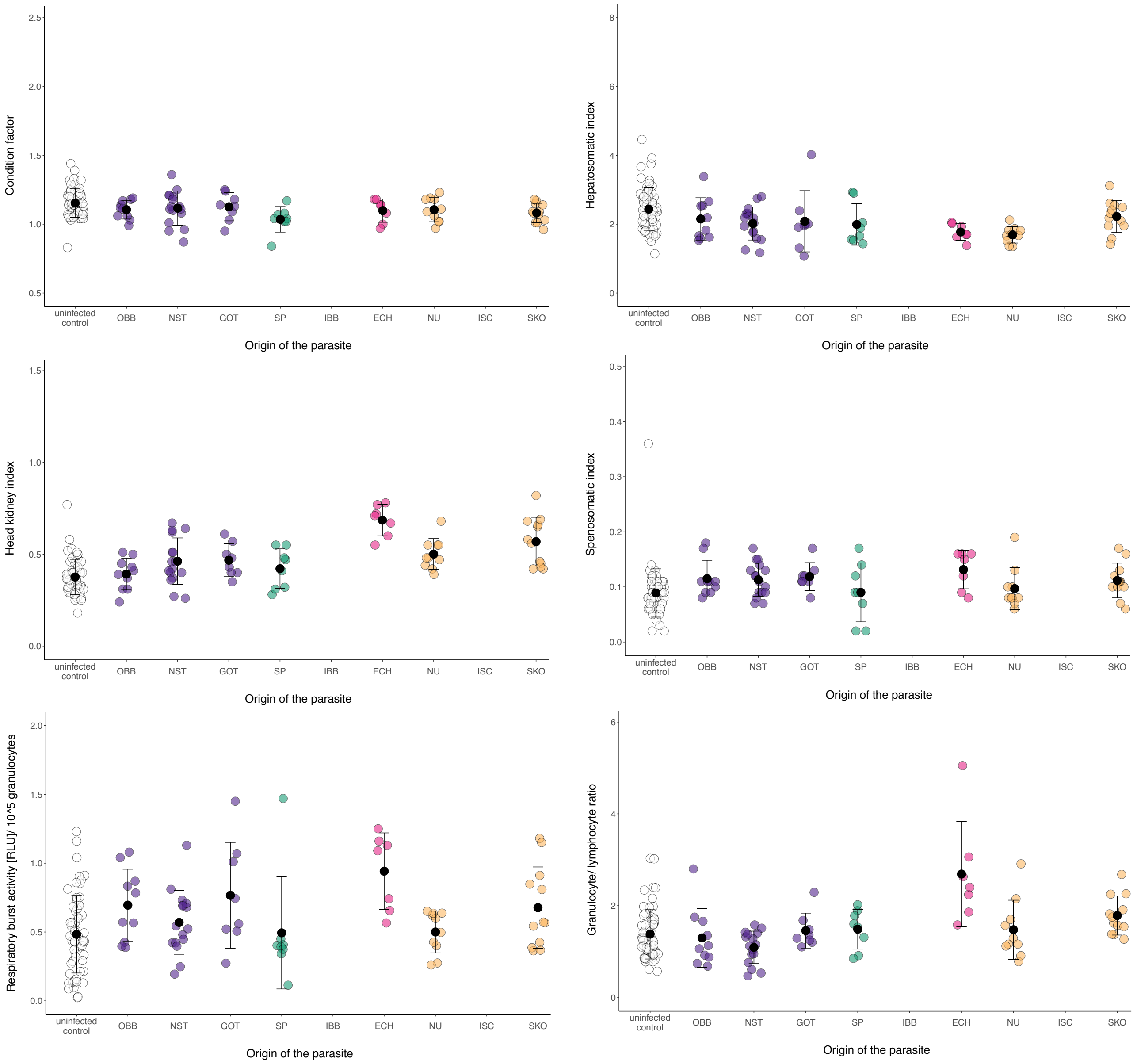

Figure S2. Phenotypic differences between sham-exposed and *S. solidus* infected DE sticklebacks (*contrast 2*).

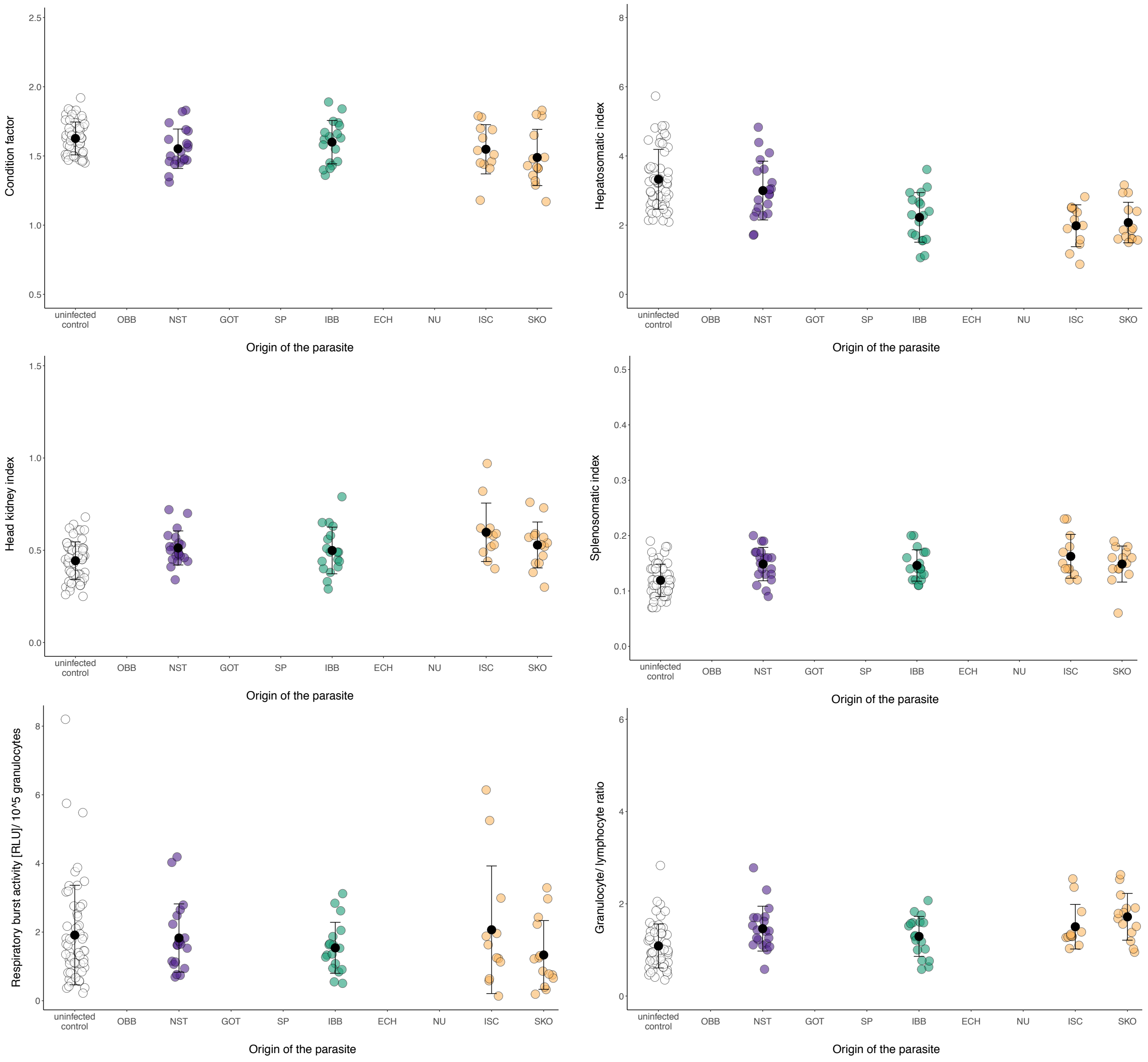

Figure S3. Phenotypic differences between sham-exposed and *S. solidus* infected DE sticklebacks (DE in *contrast 1*).

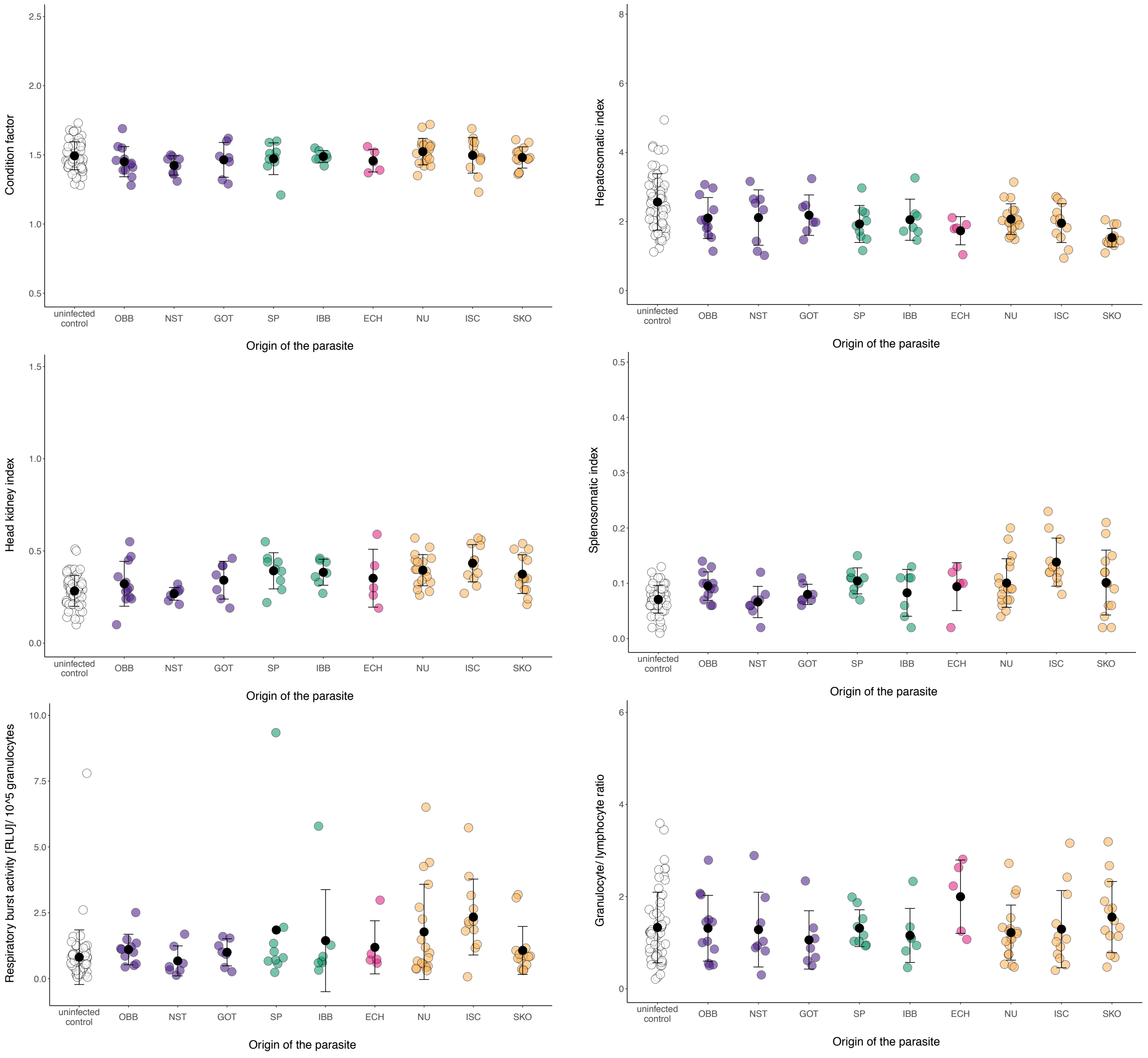

Figure S4. Phenotypic differences between sham-exposed and *S. solidus* infected NO sticklebacks (*contrast 3*).

SI.4 Supplementary information on host immune gene expression (*contrast 1*)

SI.4.1 Stickleback immune gene expression differences between populations

Table S8. Differentially expressed immune genes of sham-exposed DE and NO sticklebacks (*contrast 1*)

PERMANOVA results	Df	SumsOfSqs	F.Model	Pr(<F)	R2
all genes	1	10.226	4.4637	0.0012	0.08704
innate	1	4.249	3.3172	0.009699	0.06595
ANOVA results	numDF	denDF	F-value	p-value	pseudo R2
marco	1	44	1.034201	0.3147	0.202792
mst1ra	1	44	0.988256	0.3256	0.1088608
mif1	1	44	0.003197	0.9552	0.2315523
il-1β	1	44	0.202999	0.6545	0.04337287
tnfr1	1	44	0.7095827	0.4041	0.175504
saal1	1	44	0.7411738	0.3940	0.1033716
tlr2	1	44	0.411762	0.5244	0.1649114
csf3r	1	44	7.288786	0.0098	0.4030412
$p22^{phox}$	1	44	19.317685	**0.0001**	0.4384317
nkef-b	1	44	1.349697	0.2516	0.05610734
sla1	1	44	9.537701	**0.0035**	0.3700301
cd97	1	44	3.454024	0.0698	
adaptive	1	3.9233	6.7610	0.0018	0.12663
stat4	1	44	3.703683	0.0608	0.07364854
stat6	1	44	5.313142	0.0259	0.319497
igm	1	44	11.776301	**0.0013**	0.3308283
cd83	1	44	0.071832	0.7899	0.03668696
foxp3	1	44	0.7612114	0.3877	0.2346003
tgf-β	1	44	0.3527227	0.5556	0.127027
tcr-β	1	44	54.47217	**< 0.0001**	0.5979209
il16	1	44	2.9887492	0.0909	0.2852176
mhcII	1	44	6.409371	0.0150	0.6314256
complement	1	2.0539	4.7784	0.0039	0.09302
cfb	1	44	5.792049	0.0204	0.3207292
c7	1	44	20.639223	**< 0.0001**	0.3323971
c9	1	44	0.8890409	0.3509	0.1934673

All models are based on log10-transformed calibrated normalized relative quantities (CNRQ values) and include the weight of the fish as covariate to account for size related effects. Non-parametric permutational multivariate analyses of variance (PERMANOVA) were calculated on Euclidian distances and 10,000 permutations; permutations were constrained within 'round'. If results from multivariate statistics remained significant after FDR correction, single genes were analyzed with linear mixed models (LMMs) and analyses of variance (ANOVAs). Conditional pseudo R^2 values (Nakagawa and Schielzeth, 2013; Johnson, 2014) were calculated with the function sem.model.fits() from *piecewiseSEM* (Lefcheck, 2016). Differentially expressed genes are marked in bold letters if significant after FDR correction.

Table S9. Differentially expressed immune genes of ISC-infected DE and NO sticklebacks (*contrast 1*)

PERMANOVA results	Df	SumsOfSqs	F.Model	Pr(<F)	R2
all genes	1	6.123	2.8019	0.015798	0.10076
innate	1	4.661	3.5770	0.0044	0.12548
ANOVA results	numDF	denDF	F-value	p-value	pseudo R2
marco	1	18	0.8257869	0.3755	0.2335003
mst1ra	1	18	1.891570	0.1859	0.4391818
mif1	1	18	0.1941722	0.6647	0.2549664
il-1β	1	18	19.989167	**0.0003**	0.4332452
tnfr1	1	18	0.222879	0.6425	0.3809059
saal1	1	18	0.003260	0.9551	0.5787717
tlr2	1	18	0.032950	0.8580	0.267587
csf3r	1	18	4.140233	0.0569	0.1009758
p22^{phox}	1	18	0.284175	0.6005	0.3422705
nkef-b	1	18	3.658218	0.0718	0.3553208
sla1	1	18	0.000222	0.9883	0.3292233
cd97	1	18	0.195377	0.6637	0.2935738
adaptive	not significant after FDR correction				
complement	not significant after FDR correction				

Sticklebacks were infected with single *S. solidus* plerocercoids from an Icelandic (ISC) population. All models are based on log10-transformed calibrated normalized relative quantities (CNRQ values) and include the weight of the fish as covariate to account for size related effects. Non-parametric permutational multivariate analyses of variance (PERMANOVA) were calculated on Euclidian distances and 10,000 permutations; permutations were constrained within 'round'. If results from multivariate statistics remained significant after FDR correction, single genes were analyzed with linear mixed models (LMMs) and analyses of variance (ANOVAs). Conditional pseudo R^2 values (Nakagawa and Schielzeth, 2013; Johnson, 2014) were calculated with the function sem.model.fits() from the R package piecewiseSEM (Lefcheck, 2016). Differentially expressed genes are marked in bold letters if significant after FDR correction.

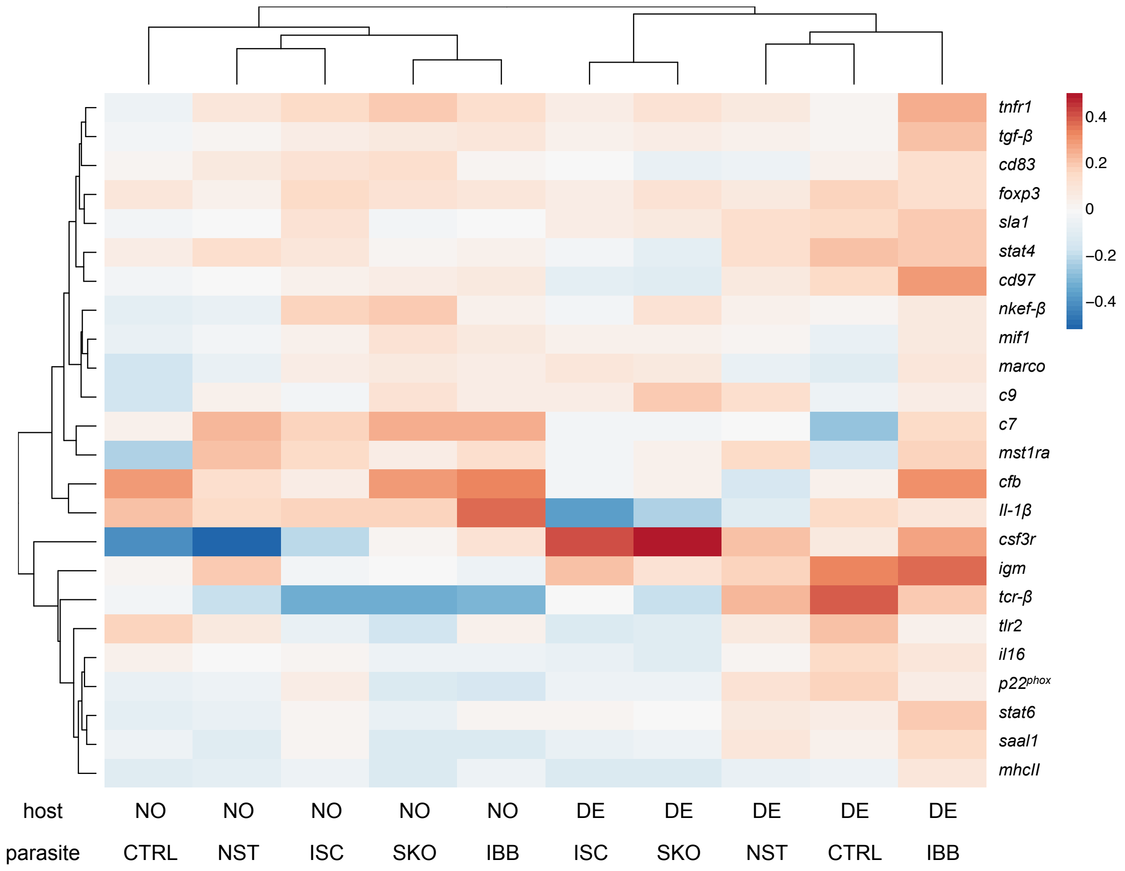

Figure S6. Heatmap showing average gene expression values in spleen samples from sham-exposed (CTRL) and *S. solidus* infected DE and NO sticklebacks. Calibrated normalized relative quantities (CNRQ) were log10-transformed. The heatmap (function aheatmap() from *NMF*) was based on Euclidian distances; rows and columns were clustered according to similarity.

SI.5 Supplementary information for *contrast 2* and *contrast 3*

Table S10. The effect of *S. solidus* origin on parasite indices in DE fish (*contrast 2*)

Explanatory		R^2	numDF	denDF	F-value	p-value
S. solidus origin		0.7841811	6	62	42.39099	< 0.0001
			Estimate	Std. Error	z-value	Pr(>|z|)
GOT	vs	ECH	-6.1425	1.0848	-5.663	<0.001
NST	vs	ECH	-7.5550	0.9467	-7.980	<0.001
NU	vs	ECH	0.1340	1.0296	0.130	1.00000
OBB	vs	ECH	-7.8000	1.0296	-7.576	<0.001
SKO	vs	ECH	2.0792	0.9936	2.093	0.35358
SP	vs	ECH	-2.0675	1.0812	-1.912	0.46838
NST	vs	GOT	-1.4125	0.9046	-1.561	0.70422
NU	vs	GOT	6.2765	0.9910	6.334	<0.001
OBB	vs	GOT	-1.6575	0.9910	-1.673	0.63143
SKO	vs	GOT	8.2217	0.9536	8.622	<0.001
SP	vs	GOT	4.0750	1.0446	3.901	0.00185
NU	vs	NST	7.6890	0.8422	9.130	<0.001
OBB	vs	NST	-0.2450	0.8422	-0.291	0.99995
SKO	vs	NST	9.6342	0.7978	12.076	<0.001
SP	vs	NST	5.4875	0.9046	6.066	<0.001
OBB	vs	NU	-7.9340	0.9343	-8.492	<0.001
SKO	vs	NU	1.9452	0.8945	2.175	0.30647
SP	vs	NU	-2.2015	0.9910	-2.222	0.28062
SKO	vs	OBB	9.8792	0.8945	11.044	<0.001
SP	vs	OBB	5.7325	0.9910	5.785	<0.001
SP	vs	SKO	-4.1467	0.9536	-4.349	< 0.001

Sticklebacks were infected with single *S. solidus* from OBB, NST, GOT, SP, ECH, NU or SKO. The linear mixed model (LMM) included ‘round’, i.e. host and parasite genotype combinations, as random intercept. The R^2 includes the effect of the random term and was calculated according to (Nakagawa and Schielzeth, 2013; Johnson, 2014; Lefcheck, 2016). Post hoc tests are based on Tukey’s all pair comparisons.

Table S11. The effect of *S. solidus* origin on parasite indices in DE fish (in *contrast 1*)

Explanatory		R^2	numDF	denDF	F-value	p-value
S. solidus origin		0.8037465	3	58	68.63429	< 0.0001
			Estimate	Std. Error	z-value	Pr(>|z|)
ISC	vs	IBB	1.5266	0.9236	1.653	0.348
NST	vs	IBB	-6.0916	0.8223	-7.408	<0.001
SKO	vs	IBB	5.9563	0.9007	6.613	<0.001
NST	vs	ISC	-7.6181	0.8934	-8.527	<0.001
SKO	vs	ISC	4.4297	0.9692	4.571	<0.001
SKO	vs	NST	12.0478	0.8638	13.947	<0.001

Sticklebacks were infected with single *S. solidus* from NST, IBB, ISC, or SKO. The linear mixed model (LMM) included ‘round’, i.e. host and parasite genotype combinations, as random intercept. The R^2 includes the effect of the random term and was calculated according to (Nakagawa and Schielzeth, 2013; Johnson, 2014; Lefcheck, 2016). Post hoc tests are based on Tukey’s all pair comparisons.

Table S12. The effect of *S. solidus* origin on parasite indices in NO sticklebacks (*contrast 3*)

Explanatory			R^2	numDF	denDF	F-value	p-value
S. solidus origin			0.6242631	8	81	61.08925	< 0.0001
				Estimate	Std. Error	z-value	Pr(>|z|)
GOT	vs	ECH		-2.99800	1.31129	-2.286	0.30948
IBB	vs	ECH		3.46771	1.32980	2.608	0.15671
ISC	vs	ECH		2.99283	1.38830	2.156	0.39017
NST	vs	ECH		-3.58425	1.23234	-2.908	0.07195
NU	vs	ECH		5.85811	1.41111	4.151	<0.001
OBB	vs	ECH		-3.67717	1.22033	-3.013	0.05374
SKO	vs	ECH		7.08414	1.46836	4.825	<0.001
SP	vs	ECH		2.18311	1.67868	1.300	0.91545
IBB	vs	GOT		6.46571	0.77373	8.357	<0.001
ISC	vs	GOT		5.99083	0.87043	6.883	<0.001
NST	vs	GOT		-0.58625	0.59072	-0.992	0.98271
NU	vs	GOT		8.85611	0.90637	9.771	<0.001
OBB	vs	GOT		-0.67917	0.56523	-1.202	0.94534
SKO	vs	GOT		10.08214	0.99316	10.152	<0.001
SP	vs	GOT		5.18111	1.28384	4.036	0.00138
ISC	vs	IBB		-0.47488	0.89808	-0.529	0.99979
NST	vs	IBB		-7.05196	0.63075	-11.180	<0.001
NU	vs	IBB		2.39040	0.93296	2.562	0.17371
OBB	vs	IBB		-7.14488	0.60694	-11.772	< 0.001
SKO	vs	IBB		3.61643	1.01748	3.554	0.00908
SP	vs	IBB		-1.28460	1.30274	-0.986	0.98338
NST	vs	ISC		-6.57708	0.74621	-8.814	<0.001
NU	vs	ISC		2.86528	1.01460	2.824	0.09069
OBB	vs	ISC		-6.67000	0.72620	-9.185	<0.001
SKO	vs	ISC		4.09131	1.09282	3.744	0.00466
SP	vs	ISC		-0.80972	1.36240	-0.594	0.99951
NU	vs	NST		9.44236	0.78784	11.985	<0.001
OBB	vs	NST		-0.09292	0.34448	-0.270	1.00000
SKO	vs	NST		10.66839	0.88631	12.037	<0.001
SP	vs	NST		5.76736	1.20309	4.794	<0.001
OBB	vs	NU		-9.53528	0.76892	-12.401	<0.001
SKO	vs	NU		1.22603	1.12166	1.093	0.96852
SP	vs	NU		-3.67500	1.38564	-2.652	0.14060
SKO	vs	OBB		10.76131	0.86953	12.376	<0.001
SP	vs	OBB		5.86028	1.19078	4.921	<0.001
SP	vs	SKO		-4.90103	1.44390	-3.394	0.01638

Sticklebacks were either sham-exposed or infected with single *S. solidus* from OBB, NST, GOT, SP, IBB, ECH, NU, ISC or SKO. The linear mixed model (LMM) included 'round', i.e. host and parasite genotype combinations, as random intercept. The R^2 was calculated according to (Nakagawa and Schielzeth, 2013; Johnson, 2014; Lefcheck, 2016). Post hoc tests are based on Tukey's all pair comparisons.

SI.5.1 Stickleback immune gene expression according to clustered localities of parasites

Table S13. The effect of parasite origin on immune gene expression in *S. solidus* infected DE sticklebacks (*contrast 2*: Pacific versus Baltic parasites)

PERMANOVA results	Df	SumsOfSqs	F.Model	Pr(<F)	R2
all genes	1	11.612	4.7401	0.0047	0.13047
innate	1	4.442	3.8777	0.0183	0.10833
ANOVA results	numDF	denDF	F-value	p-value	pseudo R2
marco	1	29	0.6705910	0.4195	0.1577212
mst1ra	1	29	0.7120762	0.4057	0.2422407
mif1	1	29	24.383022	**< 0.0001**	0.4261418
il-1β	1	29	2.646215	0.1146	0.2497382
tnfr1	1	29	19.531804	**0.0001**	0.1943971
saal1	1	29	0.6674974	0.4206	0.05301745
tlr2	1	29	0.9491798	0.3380	0.3058376
csf3r	1	29	8.230615	0.0076	0.5995144
$p22^{phox}$	1	29	0.3811838	0.5418	0.1390658
nkef-b	1	29	2.782856	0.1060	0.2846311
sla1	1	29	5.547274	0.0255	0.2100529
cd97	1	29	5.671517	0.0240	0.1500293
adaptive	1	3.5866	4.1608	0.0126	0.11695
stat4	1	29	0.1462244	0.7050	0.08385778
stat6	1	29	0.9020573	0.3501	0.04451725
igm	1	29	0.0193593	0.8903	0.4507765
cd83	1	29	1.8584893	0.1833	0.2673836
foxp3	1	29	6.688682	0.0150	0.2362648
tgf-β	1	29	2.9102652	0.0987	0.2368642
tcr-β	1	29	0.273943	0.6047	0.02215725
il16	1	29	4.212606	0.0492	0.2792412
mhcII	1	29	7.260669	0.0116	0.6589304
complement	1	3.5827	8.1042	0.0014	0.20684
cfb	1	29	6.734624	0.0147	0.1718487
c7	1	29	0.754282	0.3923	0.0116967
c9	1	29	10.791768	**0.0027**	0.347015

DE sticklebacks were infected with single *S. solidus* from the Pacific (ECH) or the Baltic region (OBB, NST, GOT). All models are based on log10-transformed calibrated normalized relative quantities (CNRQ values) and include the weight of the fish as covariate to account for size related effects. Non-parametric permutational multivariate analyses of variance (PERMANOVA) were calculated on Euclidian distances and 10,000 permutations; permutations were constrained within ‘round’. If results from multivariate statistics remained significant after FDR correction, single genes were analyzed with linear mixed models (LMMs) and analyses of variance (ANOVAs). Conditional pseudo R^2 values (Nakagawa and Schielzeth, 2013; Johnson, 2014) were calculated with the R package piecewiseSEM (Lefcheck, 2016). Differentially expressed genes are marked in bold letters if significant after FDR correction.

Table S14. The effect of parasite origin on immune gene expression in *S. solidus* infected DE sticklebacks (*contrast 2*: Pacific versus Atlantic parasites)

PERMANOVA results	Df	SumsOfSqs	F.Model	Pr(<F)	R2
all genes	1	7.483	3.7018	0.0040	0.13122
innate	not significant				
adaptive	1	4.3787	5.8377	0.0002	0.19318
ANOVA results	numDF	denDF	F-value	p-value	pseudo R2
stat4	1	22	3.183325	0.0882	0.2131196
stat6	1	22	0.8429660	0.3685	0.3147911
igm	1	22	0.173993	0.6806	0.2110768
cd83	1	22	0.8383639	0.3698	0.03927397
foxp3	1	22	6.090012	0.0218	0.2836478
tgf-β	1	22	0.2983933	0.5904	0.2660784
tcr-β	1	22	2.8142746	0.1076	0.1019166
il16	1	22	2.9249314	0.1013	0.3136332
mhcII	1	22	15.711934	**0.0007**	0.3365486
complement	1	1.6097	3.6639	0.0161	0.13192
cfb	1	22	5.561498	0.0277	0.176698
c7	1	22	0.0013976	0.9705	0.01687028
c9	1	22	2.386635	0.1366	0.2022392

DE sticklebacks were infected with single *S. solidus* from the Pacific (ECH) or the Atlantic region (NU, SKO). All models are based on log10-transformed calibrated normalized relative quantities (CNRQ values) and include the weight of the fish as covariate to account for size related effects. Non-parametric permutational multivariate analyses of variance (PERMANOVA) were calculated on Euclidian distances and 10,000 permutations; permutations were constrained within 'round'. If results from multivariate statistics remained significant after FDR correction, single genes were analyzed with linear mixed models (LMMs) and analyses of variance (ANOVAs). Conditional pseudo R^2 values (Nakagawa and Schielzeth, 2013; Johnson, 2014) were calculated with the R package piecewiseSEM (Lefcheck, 2016). Differentially expressed genes are marked in bold letters if significant after FDR correction.

Table S15. The effect of parasite origin on immune gene expression in *S. solidus* infected NO sticklebacks (*contrast* 3: Pacific versus Baltic parasites)

PERMANOVA results	Df	SumsOfSqs	F.Model	Pr(<F)	R2
all genes	1	9.896	4.0409	0.0028	0.12559
innate	1	4.562	3.2630	0.006399	0.10441
ANOVA results	numDF	denDF	F-value	p-value	pseudo R2
marco	1	25	8.392080	**0.0077**	0.2271724
mst1ra	1	25	3.77560	0.0633	0.03548697
mif1	1	25	5.541702	**0.0267**	0.6104602
il-1β	1	25	4.012346	0.0561	0.2204628
tnfr1	1	25	17.895225	**0.0003**	0.4698114
saal1	1	25	0.2236917	0.6403	0.02951859
tlr2	1	25	0.68259	0.4165	0.2310644
csf3r	1	25	3.0020583	0.0955	0.4896976
**$p22^{phox}$**	1	25	7.051231	**0.0136**	0.4252072
nkef-b	1	25	11.806990	**0.0021**	0.4074129
sla1	1	25	16.878200	**0.0004**	0.5575598
cd97	1	25	17.052705	**0.0004**	0.1673577
adaptive	1	3.6002	5.7965	0.0020	0.16799
stat4	1	25	20.719634	**0.0001**	0.3796316
stat6	1	25	2.5762096	0.1210	0.101408
igm	1	25	5.040543	0.0338	0.1895309
cd83	1	25	13.400433	**0.0012**	0.4245893
foxp3	1	25	13.907577	**0.0010**	0.4174076
tgf-β	1	25	38.95597	**< 0.0001**	0.2843239
tcr-β	1	25	0.9623782	0.3360	0.4709141
il16	1	25	20.30002	**0.0001**	0.2542416
mhcII	1	25	11.242303	**0.0025**	0.5879825
complement	not significant after FDR correction				

NO sticklebacks were infected with single *S. solidus* from the Pacific (ECH) or the Baltic region (OBB, NST, GOT). All models are based on log10-transformed calibrated normalized relative quantities (CNRQ values) and include the weight of the fish as covariate to account for size related effects. Non-parametric permutational multivariate analyses of variance (PERMANOVA) were calculated on Euclidian distances and 10,000 permutations; permutations were constrained within 'round'. If results from multivariate statistics remained significant after FDR correction, single genes were analyzed with linear mixed models (LMMs) and analyses of variance (ANOVAs). Conditional pseudo R^2 values (Nakagawa and Schielzeth, 2013; Johnson, 2014) were calculated with the R package piecewiseSEM (Lefcheck, 2016). Differentially expressed genes are marked in bold letters if significant after FDR correction.

Table S16. The effect of parasite origin on immune gene expression in *S. solidus* infected NO sticklebacks (*contrast 3*: Pacific versus Atlantic parasites)

PERMANOVA results	Df	SumsOfSqs	F.Model	Pr(<F)	R2
all genes	1	11.136	3.9272	0.0037	0.07781
innate	1	4.772	2.9472	0.0142	0.05932
ANOVA results	numDF	denDF	F-value	p-value	pseudo R2
marco	1	43	1.6216848	0.2097	0.1003356
mst1ra	1	43	10.335062	**0.0025**	0.06026265
mif1	1	43	0.24499306	0.6231	0.600834
il-1β	1	43	7.41588	**0.0093**	0.1414507
tnfr1	1	43	9.378793	**0.0038**	0.1669963
saal1	1	43	0.934775	0.3390	0.1185011
tlr2	1	43	2.2991785	0.1368	0.554773
csf3r	1	43	1.337027	0.2539	0.3369563
p22^{phox}	1	43	7.219358	**0.0102**	0.2022315
nkef-b	1	43	10.156009	**0.0027**	0.08408415
sla1	1	43	21.053776	**< 0.0001**	0.09562046
cd97	1	43	13.395692	**0.0007**	0.07031316
adaptive	1	4.269	5.2682	0.0037	0.10309
stat4	1	43	14.963542	**0.0004**	0.1379449
stat6	1	43	2.9758228	0.0917	0.07796727
igm	1	43	12.137119	**0.0011**	0.2510531
cd83	1	43	5.426821	**0.0246**	0.2126779
foxp3	1	43	7.771886	**0.0079**	0.1427779
tgf-β	1	43	15.66522	**0.0003**	0.06727398
tcr-β	1	43	0.191459	0.6639	0.2949225
il16	1	43	20.661348	**< 0.0001**	0.1248736
mhcII	1	43	8.220390	**0.0064**	0.275311
complement	1	2.0950	5.1591	0.007999	0.09852
cfb	1	43	3.007361	0.0901	0.1230495
c7	1	43	1.942404	0.1706	0.02336822
c9	1	43	17.776506	**0.0001**	0.2357195

NO sticklebacks were infected with single *S. solidus* from Pacific (ECH) or the Atlantic region (NU, ISC, SKO). All models are based on log10-transformed calibrated normalized relative quantities (CNRQ values) and include the weight of the fish as covariate to account for size related effects. Non-parametric permutational multivariate analyses of variance (PERMANOVA) were calculated on Euclidian distances and 10,000 permutations; permutations were constrained within 'round'. If results from multivariate statistics remained significant after FDR correction, single genes were analyzed with linear mixed models (LMMs) and analyses of variance (ANOVAs). Conditional pseudo R^2 values (Nakagawa and Schielzeth, 2013; Johnson, 2014) were calculated with the R package piecewiseSEM (Lefcheck, 2016). Differentially expressed genes are marked in bold letters if significant after FDR correction.

SI.5.2 Stickleback immune gene expression in infected versus control fish

ECH-infected DE sticklebacks had significantly higher expression of three genes of innate immunity, one gene of adaptive immunity (*foxp3*) and complement *c9*; RNA levels of *tcr-β* and *mhcII* were significantly lower than in controls (Table S17). NU-infected DE sticklebacks had significantly higher expression of five innate immune genes and two complement components; again, *tcr-β* was significantly lower expressed than in controls (Table S18). SKO-infected DE sticklebacks had significantly lower expression of the genes *igm* and *tcr-β* (Table S19).

In NO hosts, four genes were significantly higher expressed upon infection with the ECH strain; only RNA levels of *tlr2* were higher in controls. Infection with NU *S. solidus* was linked to lower RNA levels of *foxp3* and *tcr-β* in comparison to controls, *mhcII* RNA levels were higher (Table S20).

Table S17. The effect of Pacific (ECH) *S. solidus* infection on immune gene expression in DE sticklebacks

PERMANOVA results	Df	SumsOfSqs	F.Model	Pr(<F)	R2
all genes	1	10.968	6.6963	< 0.0001	0.17831
innate	1	5.1367	7.51	< 0.0001	0.19545
ANOVA results	numDF	denDF	F-value	p-value	pseudo R2
marco	1	28	1.3223894	0.2599	0.04482254
mst1ra	1	28	4.963628	0.0341	0.1346899
mif1	1	28	22.335106	**0.0001**	0.4110788
il-1β	1	28	4.135334	0.0516	0.4828812
tnfr1	1	28	38.28464	**< 0.0001**	0.5536073
saal1	1	28	0.9519854	0.3376	0.04472742
tlr2	1	28	2.4176792	0.1312	0.05212668
csf3r	1	28	39.81470	**< 0.0001**	0.5984884
$p22^{phox}$	1	28	0.014436	0.9052	0.09826987
nkef-b	1	28	4.096781	0.0526	0.1156389
sla1	1	28	5.463977	0.0268	0.1726114
cd97	1	28	3.979375	0.0559	0.2699205
adaptive	1	3.7237	6.4701	0.0006	0.17312
stat4	1	28	0.7173548	0.4042	0.1566161
stat6	1	28	2.750128	0.1084	0.1536997
igm	1	28	0.577116	0.4538	0.05510709
cd83	1	28	0.8975855	0.3515	0.04130682
foxp3	1	28	10.969312	**0.0026**	0.3648484
tgf-β	1	28	6.312335	0.0180	0.1654147
tcr-β	1	28	20.170149	**0.0001**	0.6763045
il16	1	28	5.640592	0.0246	0.1530182
mhcII	1	28	7.095925	**0.0127**	0.6559287
complement	1	2.11	5.5693	0.007	0.15362
cfb	1	28	1.0761172	0.3084	0.05403997
c7	1	28	6.37071	0.0176	0.2160032
c9	1	28	15.702800	**0.0005**	0.3294452

DE sticklebacks were infected with single *S. solidus* from the Pacific region (ECH) or sham-exposed as controls. All models are based on log10-transformed calibrated normalized relative quantities (CNRQ values) and include the weight of the fish as covariate to account for size related effects. If results from multivariate statistics remained significant after FDR correction, single genes were analyzed with linear mixed models (LMMs) and analyses of variance (ANOVAs). Conditional pseudo R^2 values (Nakagawa and Schielzeth, 2013; Johnson, 2014) were calculated with the function sem.model.fits() from *piecewiseSEM* (Lefcheck, 2016). Differentially expressed genes are marked in bold letters if significant after FDR correction.

Table S18. The effect of Atlantic (NU) *S. solidus* infection on immune gene expression in DE sticklebacks

PERMANOVA results	Df	SumsOfSqs	F.Model	Pr(<F)	R2
all genes	1	6.3423	4.5438	0.0022	0.11700
innate	1	3.2598	4.8912	0.0034	0.12816
ANOVA results	numDF	denDF	F-value	p-value	pseudo R2
marco	1	31	21.277178	**0.0001**	0.2247061
mst1ra	1	31	41.52767	**< 0.0001**	0.3802244
mif1	1	31	2.8351053	0.1023	0.07494536
il-1β	1	31	1.3225529	0.2589	0.4956676
tnfr1	1	31	17.84915	**0.0002**	0.3553141
saal1	1	31	1.1572809	0.2903	0.03720579
tlr2	1	31	0.1355171	0.7153	0.171688
csf3r	1	31	9.019195	**0.0052**	0.3367392
p22^{phox}	1	31	0.0842680	0.7735	0.1051056
nkef-b	1	31	0.2753085	0.6035	0.1033829
sla1	1	31	0.5254048	0.4740	0.215329
cd97	1	31	3.792486	0.0606	0.1558379
adaptive	1	1.817	3.925	0.009	0.0983
stat4	1	31	0.1487538	0.7024	0.2565489
stat6	1	31	5.427391	0.0265	0.225471
igm	1	31	0.002153	0.9633	0.003609847
cd83	1	31	1.2962914	0.2636	0.3051214
foxp3	1	31	0.122438	0.7288	0.1561726
tgf-β	1	31	6.105449	0.0192	0.1567034
tcr-β	1	31	19.427037	**0.0001**	0.6016856
il16	1	31	0.0135641	0.9080	0.1486146
mhcII	1	31	4.943850	0.0336	0.2853142
complement	1	1.2655	4.7503	0.0140	0.12308
cfb	1	31	0.317478	0.5772	0.01028082
c7	1	31	13.294900	**0.0010**	0.3283917
c9	1	31	9.197163	**0.0049**	0.3806867

DE sticklebacks were infected with single *S. solidus* from Scotland (NU) or sham-exposed as controls. All models are based on log10-transformed calibrated normalized relative quantities (CNRQ values) and include the weight of the fish as covariate to account for size related effects. If results from multivariate statistics remained significant after FDR correction, single genes were analyzed with linear mixed models (LMMs) and analyses of variance (ANOVAs). Conditional pseudo R^2 values (Nakagawa and Schielzeth, 2013; Johnson, 2014) were calculated with the function sem.model.fits() from *piecewiseSEM* (Lefcheck, 2016). Differentially expressed genes are marked in bold letters if significant after FDR correction.

Table S19. The effect of Atlantic (SKO) *S. solidus* infection on immune gene expression in DE sticklebacks

PERMANOVA results	Df	SumsOfSqs	F.Model	Pr(<F)	R2
all genes	1	8.252	4.8340	0.0016	0.12456
adaptive	1	4.7356	8.7604	< 0.0001	0.20144
ANOVA results	numDF	denDF	F-value	p-value	pseudo R2
stat4	1	31	6.408154	0.0166	0.3467709
stat6	1	31	1.371914	0.2504	0.1504773
igm	1	31	21.017566	**0.0001**	0.3755734
cd83	1	31	4.057735	0.0527	0.1049793
foxp3	1	31	0.6079386	0.4415	0.3040527
tgf-β	1	31	1.734336	0.1975	0.05518428
tcr-β	1	31	80.30430	**< 0.0001**	0.7823056
il16	1	31	0.01339549	0.9086	0.008679018
mhcII	1	31	4.254542	0.0476	0.2047914
complement	1	1.2540	3.4196	0.0278	0.09219
cfb	1	31	5.623478	0.0241	0.1556643
c7	1	31	1.80347	0.1890	0.0803123
c9	1	31	0.1222875	0.7289	0.3124093

DE sticklebacks were infected with single *S. solidus* from Norway (SKO) or sham-exposed as controls. All models are based on log10-transformed calibrated normalized relative quantities (CNRQ values) and include the weight of the fish as covariate to account for size related effects. If results from multivariate statistics remained significant after FDR correction, single genes were analyzed with linear mixed models (LMMs) and analyses of variance (ANOVAs). Conditional pseudo R^2 values (Nakagawa and Schielzeth, 2013; Johnson, 2014) were calculated with the function sem.model.fits() from *piecewiseSEM* (Lefcheck, 2016). Differentially expressed genes are marked in bold letters if significant after FDR correction.

Table S20. The effect of *S. solidus* infection on immune gene expression in NO sticklebacks

**CTRL vs ECH**					
PERMANOVA results	Df	SumsOfSqs	F.Model	Pr(<F)	R2
all genes	1	13.034	5.7731	0.006799	0.18870
innate	1	8.038	5.4255	0.0118	0.18141
ANOVA results	numDF	denDF	F-value	p-value	pseudo R2
marco	1	22	7.480525	**0.0121**	0.6146342
mst1ra	1	22	55.38068	**< 0.0001**	0.420314
mif1	1	22	1.7377567	0.2010	0.6540345
il-1β	1	22	4.584579	0.0436	0.1603913
tnfr1	1	22	7.089261	**0.0142**	0.6098981
saal1	1	22	0.03122978	0.8613	0.3679037
tlr2	1	22	7.701943	**0.0110**	0.2455484
csf3r	1	22	0.7428609	0.3980	0.6157382
$p22^{phox}$	1	22	6.769916	0.0163	0.2206684
nkef-b	1	22	3.1618774	0.0892	0.402492
sla1	1	22	4.423985	0.0471	0.3688205
cd97	1	22	0.0128443	0.9108	0.7624587
complement	1	7.0196	7.6098	0.007799	0.23142
cfb	1	22	1.338124	0.2598	0.1066028
c7	1	22	6.080528	0.0219	0.26121
c9	1	22	11.663511	**0.0025**	0.3391339

**CTRL vs NU**					
all genes	1	7.162	2.77725	0.008699	0.06827
adaptive	1	3.9791	5.7140	0.0016	0.13059
ANOVA results	numDF	denDF	F-value	p-value	pseudo R2
stat4	1	35	4.555951	0.0399	0.1020942
stat6	1	35	1.708545	0.1997	0.0958445
igm	1	35	4.462372	0.0419	0.5772781
cd83	1	35	3.0748604	0.0883	0.5380313
foxp3	1	35	12.229478	**0.0013**	0.3642791
tgf-β	1	35	0.00923769	0.9240	0.150044
tcr-β	1	35	18.261184	**0.0001**	0.3470945
il16	1	35	2.0088023	0.1652	0.1248851
mhcII	1	35	38.17083	**< 0.0001**	0.5933614

NO sticklebacks were infected with single *S. solidus* from the Pacific region (ECH) or Scotland (NU) or sham-exposed as controls (CTRL). All models are based on log10-transformed calibrated normalized relative quantities (CNRQ values) and include the weight of the fish as covariate to account for size related effects. If results from multivariate statistics remained significant after FDR correction, single genes were analyzed with linear mixed models (LMMs) and analyses of variance (ANOVAs). Conditional pseudo R^2 values (Nakagawa and Schielzeth, 2013; Johnson, 2014) were calculated with the function sem.model.fits() from *piecewiseSEM* (Lefcheck, 2016). Differentially expressed genes are marked in bold letters if significant after FDR correction.

SI.5.3 NMDS: infected versus control DE sticklebacks (contrast 2)

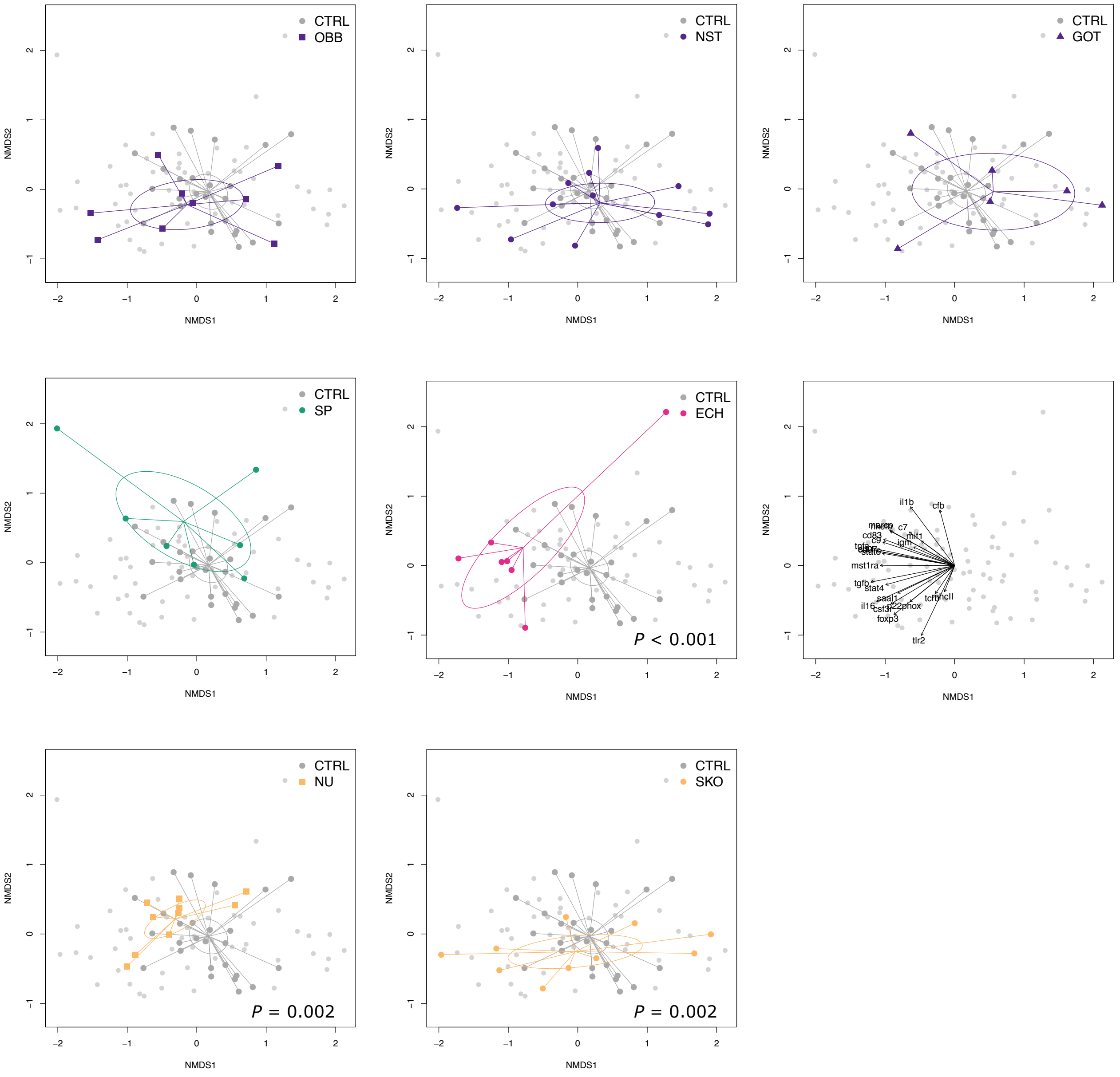

Figure S7. Non-metric multidimensional scaling (NMDS) plots on Euclidian distances and two dimensions showing multivariate data from 24 immune genes of infected and sham-exposed DE sticklebacks (2014). Each dot represents one individual; data from infected fish is colored. Ellipses represent 95% confidence intervals. *P*-values are shown if significant after FDR-correction.

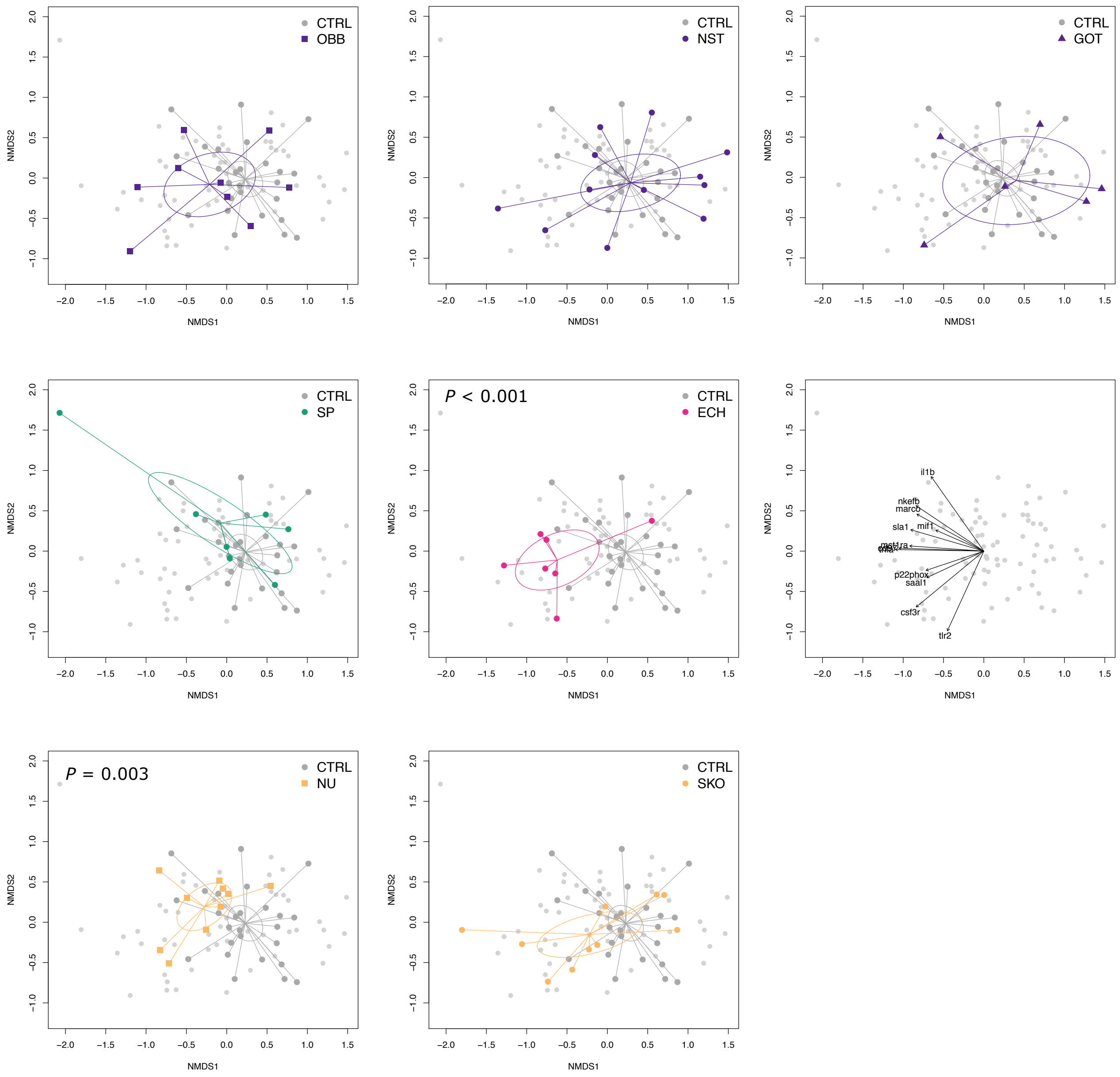

Figure S8. Non-metric multidimensional scaling (NMDS) plots on Euclidian distances and two dimensions showing multivariate data from 12 innate immune genes of infected and sham-exposed (CTRL) DE sticklebacks (2014). Each dot represents one individual; data from infected fish is colored. Ellipses represent 95% confidence intervals. *P*-values are shown if significant after FDR-correction.

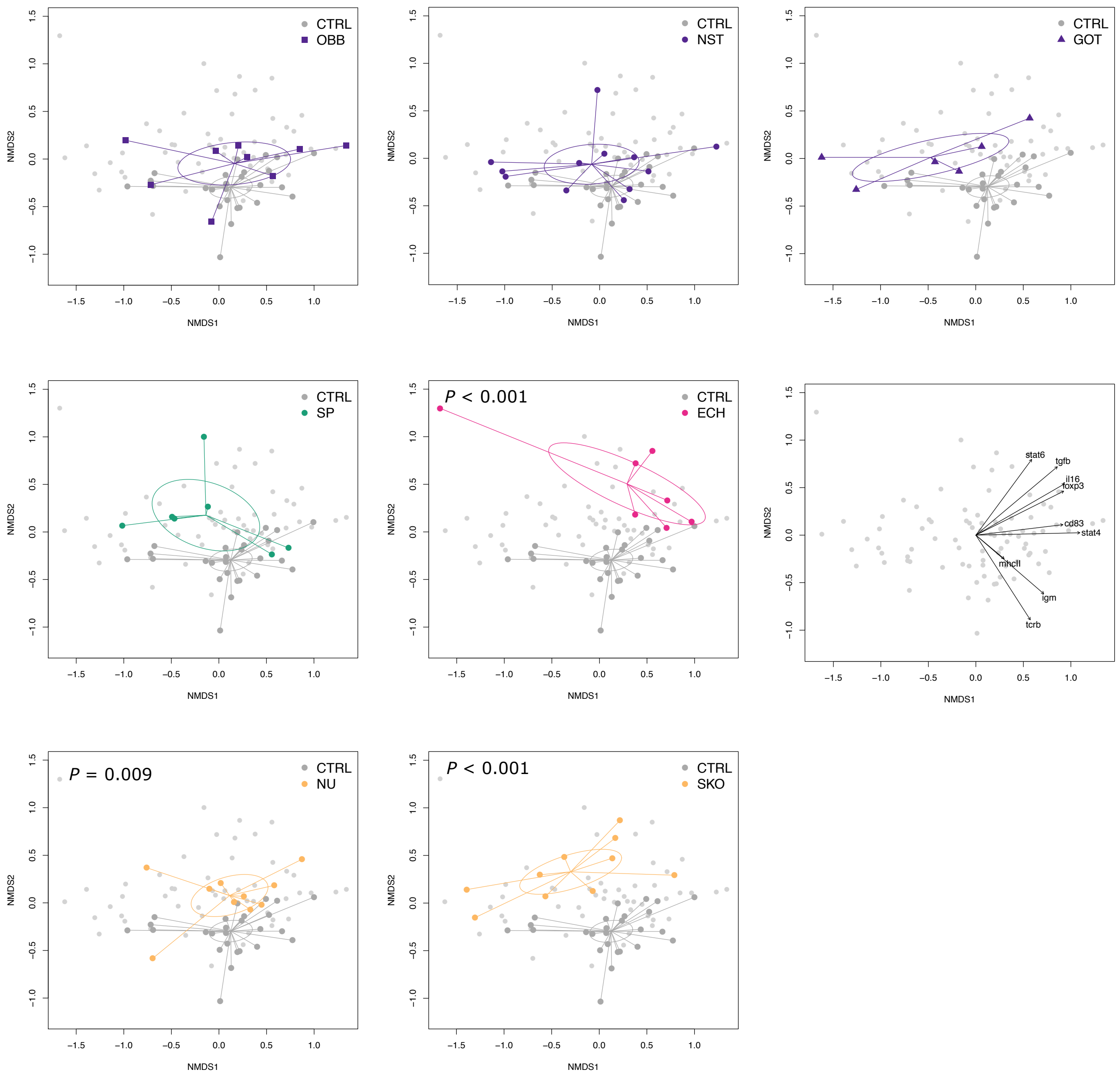

Figure S9. Non-metric multidimensional scaling (NMDS) plots on Euclidian distances and two dimensions showing multivariate data from 9 genes of the adaptive immune system of infected and sham-exposed (CTRL) DE sticklebacks (2014). Each dot represents one individual; data from infected fish is colored. Ellipses represent 95% confidence intervals. *P*-values are shown if significant after FDR-correction.

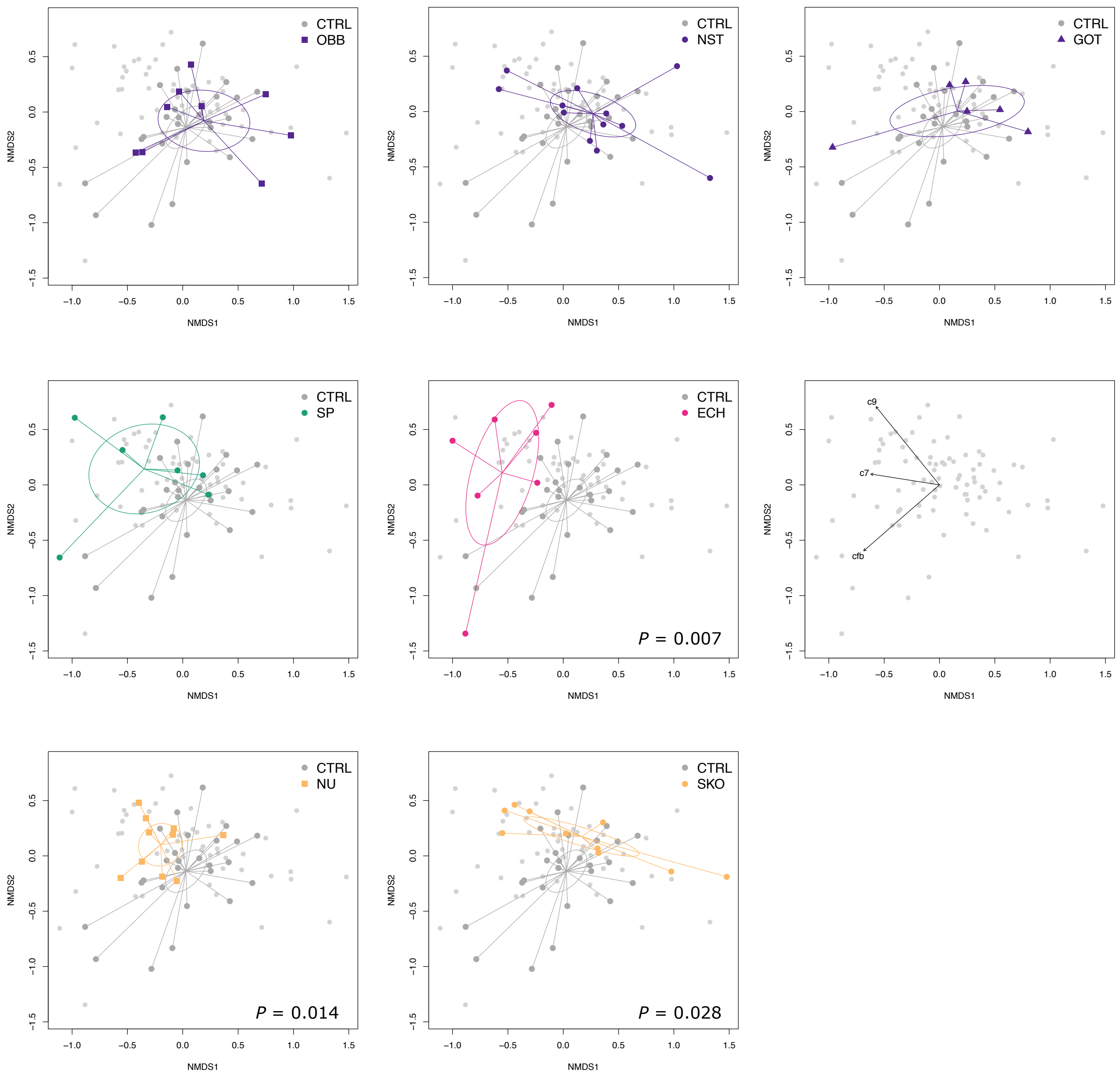

Figure S10. Non-metric multidimensional scaling (NMDS) plots on Euclidian distances and two dimensions showing multivariate data from three genes of the complement system of infected and sham-exposed DE sticklebacks (2014). Each dot represents one individual; data from infected fish is colored. Ellipses represent 95% confidence intervals. *P*-values are shown if significant after FDR-correction.

SI.5.4 NMDS: infected versus control NO sticklebacks (contrast 3)

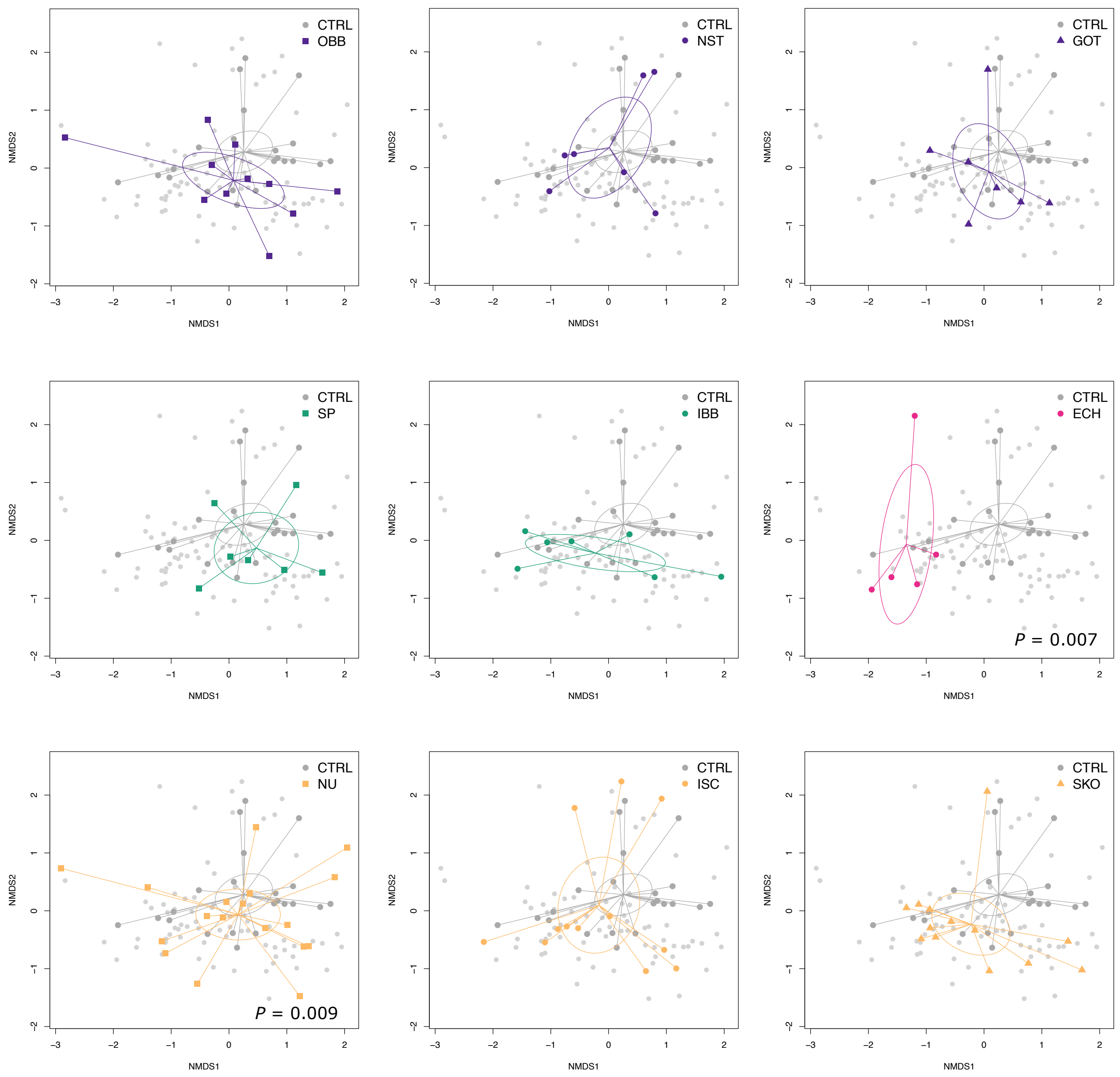

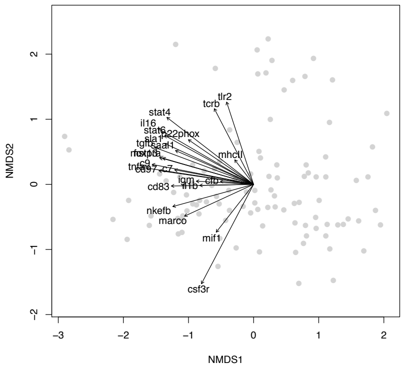

Figure S11. Non-metric multidimensional scaling (NMDS) on Euclidian distances and two dimensions showing multivariate data from 24 immune genes of infected and sham-exposed (CTRL) NO sticklebacks. Each dot represents one individual; data from infected fish is colored. Ellipses represent 95% confidence intervals. *P*-values are shown if significant after FDR-correction.

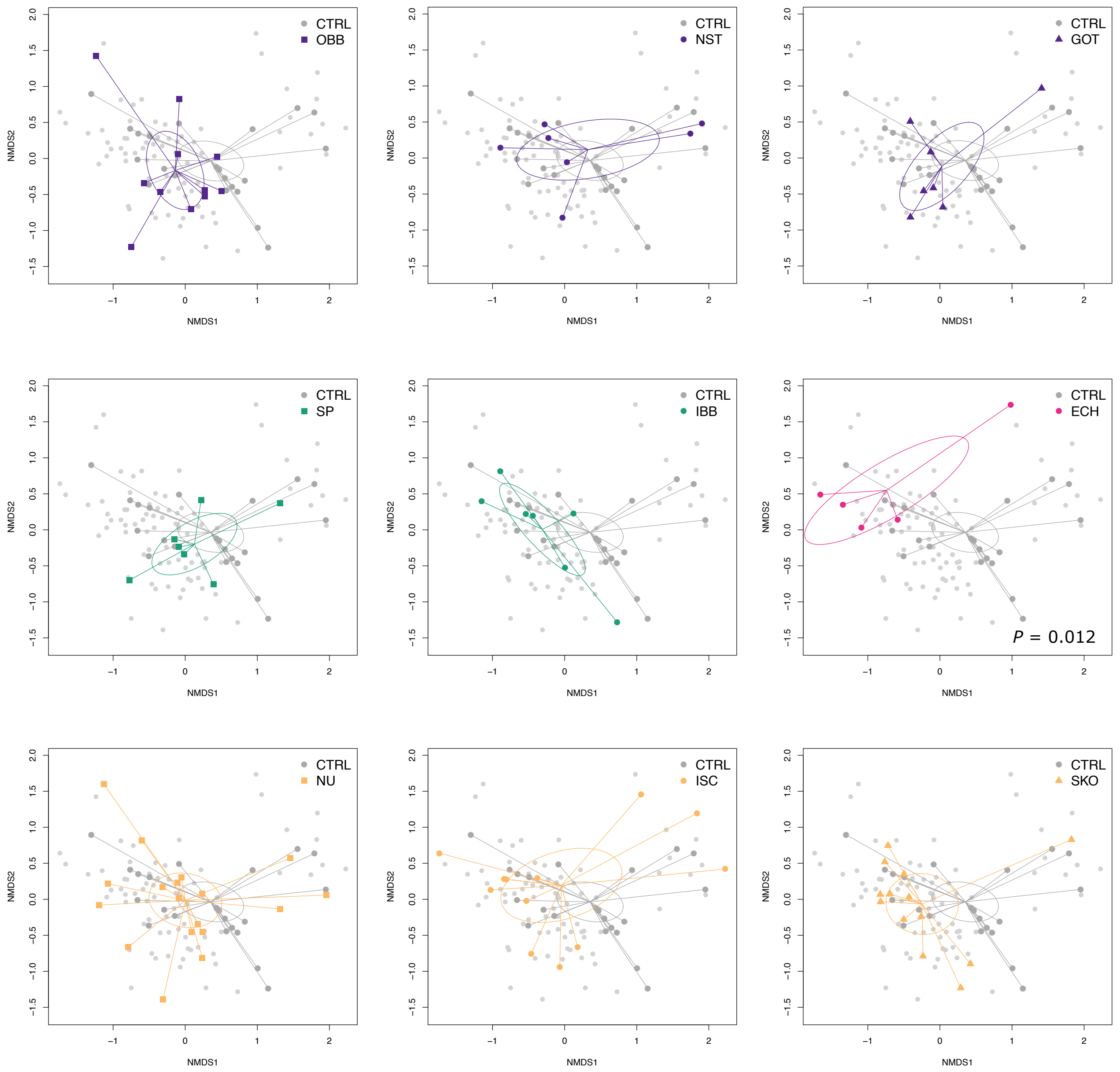

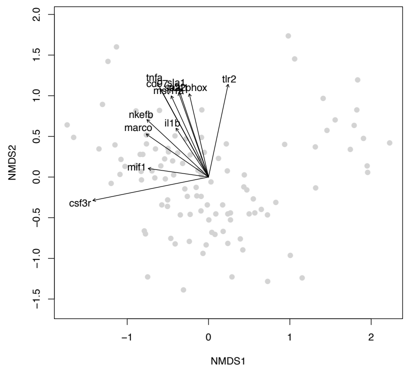

Figure S12. Non-metric multidimensional scaling (NMDS) on Euclidian distances and two dimensions showing multivariate data from 12 innate immune genes of infected and sham-exposed (CTRL) NO sticklebacks. Each dot represents one individual; data from infected fish is colored. Ellipses represent 95% confidence intervals. *P*-values are shown if significant after FDR-correction.

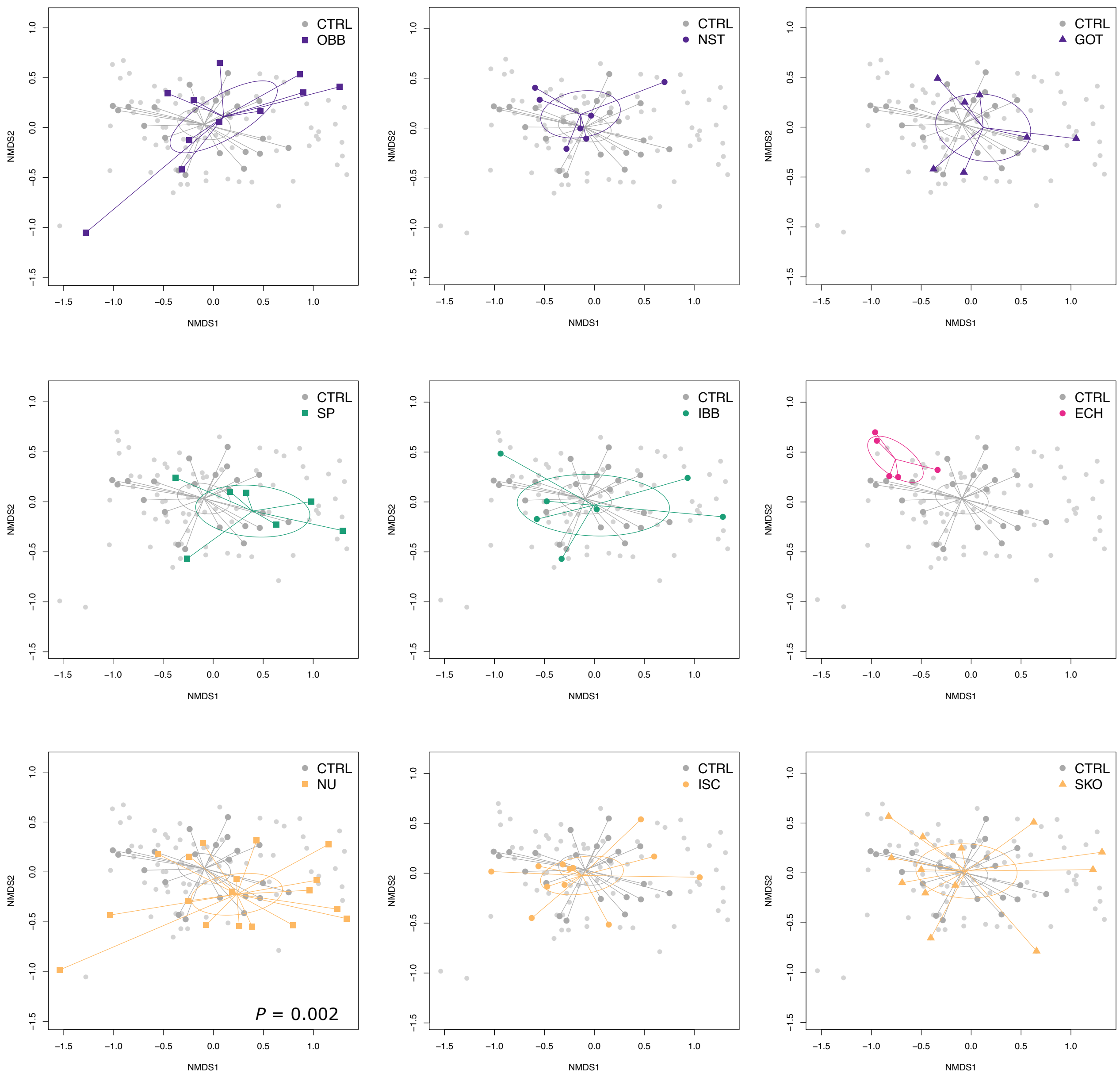

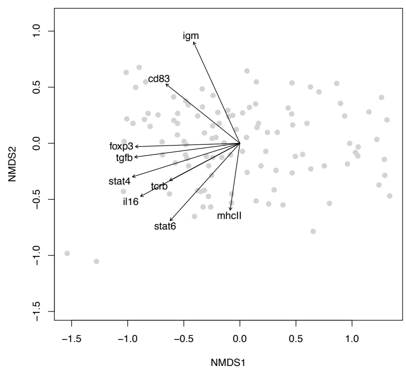

Figure S13. Non-metric multidimensional scaling (NMDS) on Euclidian distances and two dimensions showing multivariate data from 9 adaptive immune genes of infected and sham-exposed (CTRL) NO sticklebacks. Each dot represents one individual; data from infected fish is colored. Ellipses represent 95% confidence intervals. *P*-values are shown if significant after FDR-correction.

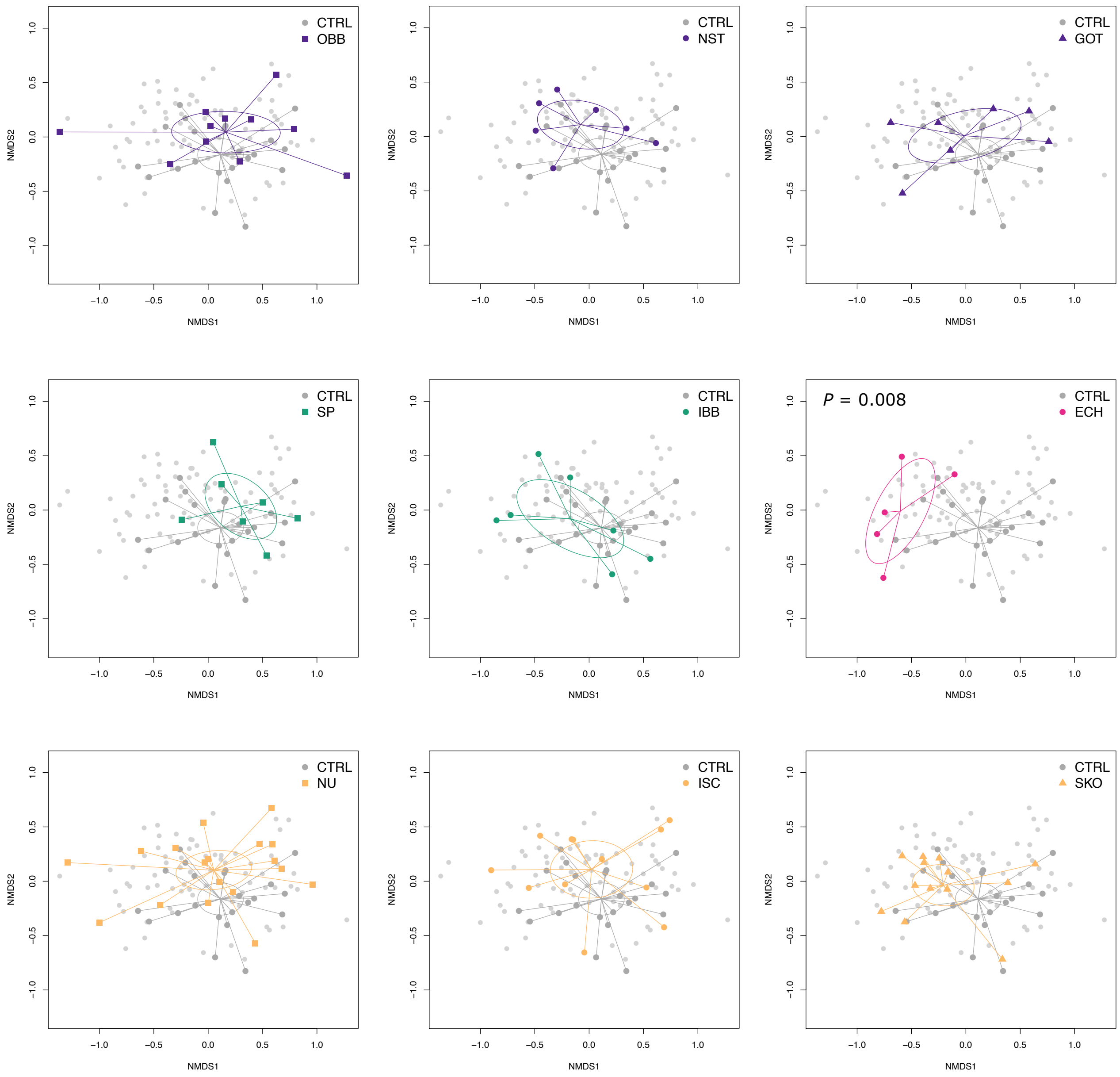

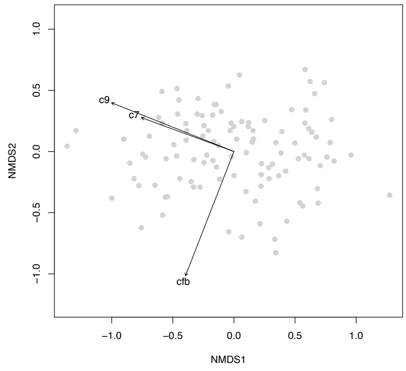

Figure S14. Non-metric multidimensional scaling (NMDS) on Euclidian distances and two dimensions showing multivariate data from three genes of the complement system of infected and sham-exposed NO sticklebacks. Each dot represents one individual; data from infected fish is colored. Ellipses represent 95% confidence intervals. *P*-values are shown if significant after FDR-correction.

SI.5.5 NMDS: infected versus control DE sticklebacks (in contrast 1*)*

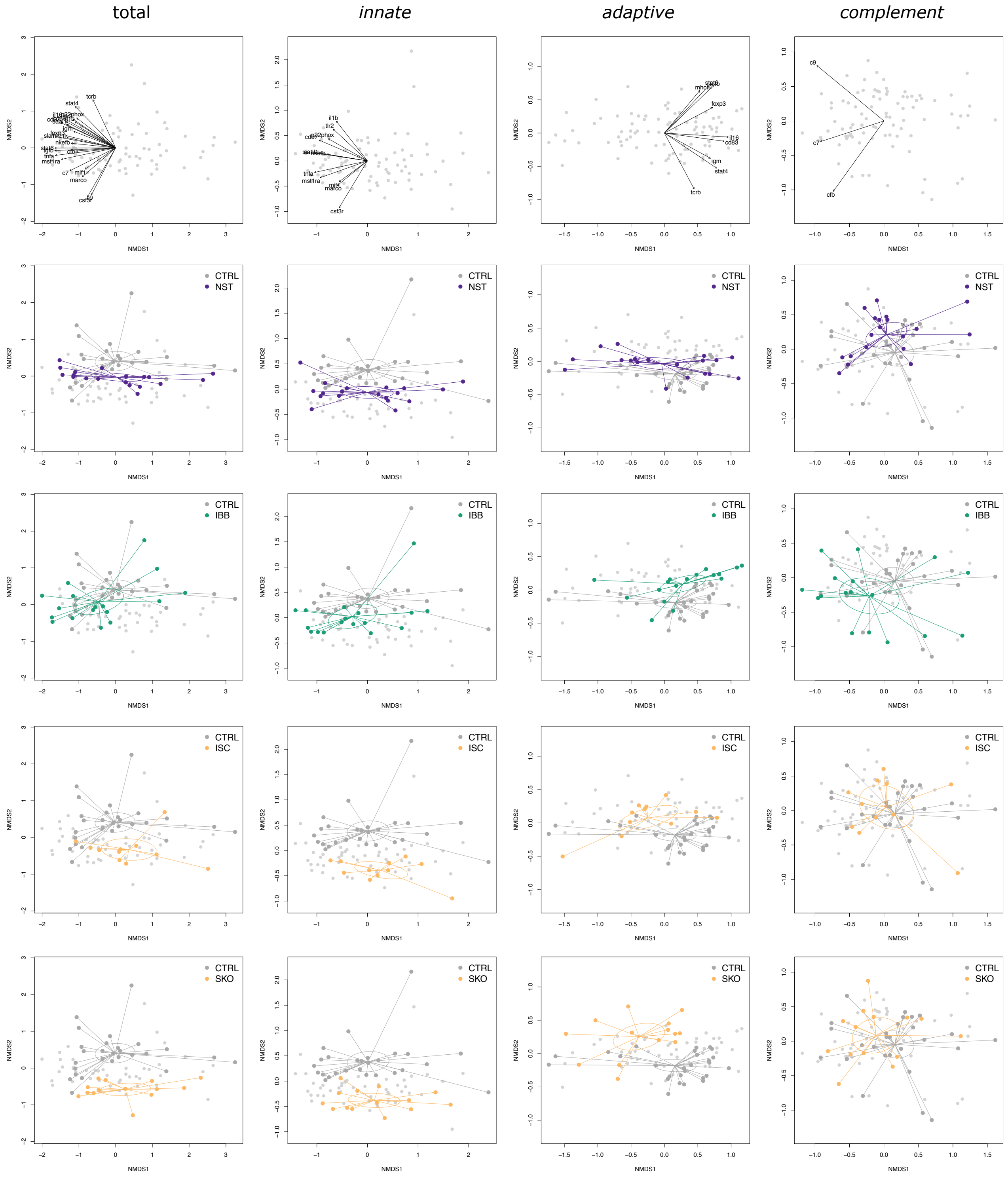

Figure S15. Non-metric multidimensional scaling (NMDS) plots on Euclidian distances and two dimensions showing multivariate data from infected and sham-exposed (CTRL) DE sticklebacks (2015). NMDS were based on log10-transformed CNRQ values of all 24 immune genes, 12 genes of the innate immune system (*marco*, *mst1ra*, *mif*, *il-1β*, *tnf-a*, *saal1*, *tlr2, csf3r*, *p22*^{phox}, *nkef-b, sla1, cd97*), 9 genes of the adaptive immune system (*stat4*, *stat6*, *igm*, *cd83*, *foxp3*, *tgf-β, il-16, mhcII, tcr-β*), or three genes of the complement system (*cfb, c7, c9*). Each dot represents one individual; colors refer to the origin of *S. solidus* in infected fish. Ellipses represent 95% confidence intervals. *P*-values are not indicated, because none were significant after FDR-correction.

References

Bates, D., Mächler, M., Bolker, B., and Walker, S. (2014). Fitting Linear Mixed-Effects Models using lme4. ArXiv14065823 Stat.

Chellappa, S., Huntingford, F.A., Strang, R.H.C., and Thomson, R.Y. (1995). Condition factor and hepatosomatic index as estimates of energy status in male three-spined stickleback. J. Fish Biol. *47*, 775–787.

Frischknecht, M. (1993). The breeding colouration of male three-spined sticklebacks (Gasterosteus aculeatus) as an indicator of energy investment in vigour. Evol. Ecol. *7*, 439–450.

Hammerschmidt, K., and Kurtz, J. (2005). Evolutionary implications of the adaptation to different immune systems in a parasite with a complex life cycle. Proc. R. Soc. B Biol. Sci. *272*, 2511–2518.

Hothorn, T., Bretz, F., and Westfall, P. (2008). Simultaneous inference in general parametric models. Biom. J. *50*, 346–363.

Johnson, P.C.D. (2014). Extension of Nakagawa & Schielzeth's R2GLMM to random slopes models. Methods Ecol. Evol. *5*, 944–946.

Kurtz, J., Kalbe, M., Aeschlimann, P.B., Häberli, M.A., Wegner, K.M., Reusch, T.B.H., and Milinski, M. (2004). Major histocompatibility complex diversity influences parasite resistance and innate immunity in sticklebacks. Proc. R. Soc. Lond. B Biol. Sci. *271*, 197–204.

Lefcheck, J.S. (2016). piecewiseSEM: Piecewise structural equation modelling in r for ecology, evolution, and systematics. Methods Ecol. Evol. *7*, 573–579.

Nakagawa, S., and Schielzeth, H. (2013). A general and simple method for obtaining R2 from generalized linear mixed-effects models. Methods Ecol. Evol. *4*, 133–142.

Pechhold, K., Pohl, T., and Kabelitz, D. (1994). Rapid quantification of lymphocyte subsets in heterogeneous cell populations by flow cytometry. Cytometry *16*, 152–159.

Scharsack, J.P., Kalbe, M., Derner, R., Kurtz, J., and Milinski, M. (2004). Modulation of granulocyte responses in three-spined sticklebacks Gasterosteus aculeatus infected with the tapeworm Schistocephalus solidus. Dis. Aquat. Organ. *59*, 141–150.

Scott, A.L., and Klesius, P.H. (1981). Chemiluminescence: A novel analysis of phagocytosis in fish. Dev. Biol. Stand. *49*, 243–254.

Seppänen, E., Kuukka, H., Voutilainen, A., Huuskonen, H., and Peuhkuri, N. (2009). Metabolic depression and spleen and liver enlargement in juvenile Arctic charr Salvelinus alpinus exposed to chronic parasite infection. J. Fish Biol. *74*, 553–561.

Wedekind, C., and Milinski, M. (1996). Do three-spined sticklebacks avoid consuming copepods, the first intermediate host of Schistocephalus solidus? — an experimental analysis of behavioural resistance. Parasitology *112*, 371–383.
