# Supplementary material for: Specificity of resistance and geographic patterns of virulence in a vertebrate host-parasite system
Source: BMC Evol Biol. 2019 Mar 19;19:80. doi: 10.1186/s12862-019-1406-3 (PMC6425677; doi:10.1186/s12862-019-1406-3)
Supplement: Supplementary file 2 — Primer information. The primer information is cited from Piecyk, Ritter & Kalbe (in review). (PDF 108 kb) [file 12862_2019_1406_MOESM2_ESM.pdf]

Gene	Function	References	ENSEMBL ID	Forward primer	Reverse primer
Reference genes					
b2m	Beta-2-microglobulin	(Hibbeler et al., 2008)	ENSGACT00000025544	GAAGATGTGTTGAATAGAAGCTGG	GAAGATGTGTTGAATAGAAGCTGG
ef1a	Elongation factor 1 α	(Hibbeler et al., 2008)	ENSGACT00000002893	CCACCGTTGCCTTTGTCC	TGGGACTGTTCCAATACCTCC
rpl13a	L13A ribosomal binding protein	(Hibbeler et al., 2008)	ENSGACT00000012319	CACCTTGGTCAACTTGAACAGTG	TCCCTCCGCCCTACGAC
ubc	Ubiquitin	(Hibbeler et al., 2008)	ENSGACT00000010662	AGACGGGCATAGCACTTGC	CAGGACAAGGAAGGCATCC
Innate					
cd97	Promotor of granulocyte and neutrophil migration, required for activation of the innate immune response	(Leemans et al., 2004; Rhodes et al., 2009)	ENSGACT00000024871	CTCGTGGCACTCTACGACATGAAG	CAGCCCTATCTTGGTGACCAGTTG
csf3r	Granulocyte colony-stimulating factor 3 receptor; role in differentiation and proliferation of granulocytes	(Birrer et al., 2012; Brunner, 2016; Maxson et al., 2013; Tabbara, 1993)	ENSGACT00000018254	TCGGGATTCGTCCTCTTCTCAG	TGGGTCAAACTTGGCTGCAC
il-1β	Interleukin 1 β; cytokine with function in early response proinflammatory signaling	(Brunner et al., 2017; Zhu et al., 2013)	ENSGACT00000019325	TGACGATGAAGCAGGTGGTCAAC	ACAGCGTCACGATCTCCTCTTC
marco, RON	Macrophage receptor with collagenous structure; mediates macrophage recognition and clearance of pathogens	(Kissick et al., 2014; Kraal et al., 2000)	ENSGACT00000001965	CCCTTTCGACCTTCACTGCC	TGTTTACCCCAACCCCTCCA

Gene	Function	References	ENSEMBL ID	Forward primer	Reverse primer
Innate (continued)					
mif1	Macrophage migration inhibitory factor; stops random macrophage migration through tissue, proinflammatory mediator of the innate immune system	(Brunner et al., 2017; Calandra and Roger, 2003)	ENSGACT00000023656	ATCAGCGGAGCTCACAACAAGC	TCAGGAGAGATGCTCAGGTGTTTG
mst1ra	Macrophage stimulating 1 receptor a; plays an important role in macrophage regulation	(Huang et al., 2016; Wang et al., 2002)	ENSGACT00000013997	ATGGCCATCGAAAGCTTGCA	TGATGTCGTACGGGTCCACA
nkef-β, peroxiredoxin 1	Natural killer cell enhancing factor; enhances cytotoxicity of NK cells, also protects against oxidative damage	(Shau et al., 1993; Stutz et al., 2015)	ENSGACT00000021380	ACTTCTCCCACTTTGCATGG	CAATGCCTTCATCCTCCTTC
p22^{phox}	NADPH oxidase component p22phox; part of the reactive oxygen species production machinery	((Bedard and Krause, 2007; Mayumi et al., 2008)	ENSGACT00000021084	GCCTCGGGACTCATTCTCCT	TGGCCCTCTTGCTTCTTGGA
saal1	Serum amyloid A; acute phase protein during inflammation response, mediates release of TNF-α and IL-1β	(Brunner et al., 2017; Haarder et al., 2013; Kovacevic et al., 2015)	ENSGACT00000007599	TCGCAGTGAGGCCAAAGATGAG	AAATCTGCCACCGTGTCCTTGG
sla1	Src-like-adaptor; necessary for maturation and activation of monocytic and dendritic cells, functions in T-cell signaling and B-cell development and function	(Brunner et al., 2017; Marton et al., 2015)	ENSGACT00000007895	ACAGAGTCGGCTCCTTCATGATAC	TCACAGAGAGCGAATACAGACCTC

Gene	Function	References	ENSEMBL ID	Forward primer	Reverse primer
Innate (continued)					
tnfr1	Tumor necrosis factor receptor 1; functions in regulation of inflammation, mediates cellular apoptosis and differentiation	(Brunner et al., 2017; Zhu et al., 2013)	ENSGACT00000013502	AACTACTACAGAGCCAAGGGCAAG	ACGGCACTCAGCGGTACAATTC
Adaptive					
cd83	Marker for mature dendritic cells, expressed on activated B and T cells, costimulatory to activate naïve and memory T-cells	(Aerts-Toegaert et al., 2007; Stutz et al., 2015)	ENSGACT00000000428	AGGACCCAGCGTATAAATGG	CCCTGGTGATTTTCCTCATC
foxp3; forkhead box N2b	Transcription factor; regulates functions important for the establishment of the T-reg lineage, key mediator of T-cell activation	(Kasheta et al., 2017; Rao and Naqvi, 2011; Robertson et al., 2015)	ENSGACT00000007261	GTTGACCCATGCAATTCCGA	CTGCTGTAGTTGTGGTCCTG
igm	Immunoglobulin heavy constant mu (IgM); antibody molecule, part of the humoral immune response	(S. Hibbeler, unpublished; Rønneseth et al., 2015; Zhu et al., 2013)	ENSGACT00000016907	AAGGCAGGAGAATGAAACCTTGG	CCGAGTGAGCAGACAGGGACTGG
il-16	Interleukin 16; cytokine with function in T-cell migration and expansion, chemoattractant for monocytes and eosinophils	(Brunner et al., 2017; Wen et al., 2006; Zhu et al., 2013)	ENSGACT00000016499	CTGGTCTGGGCTTCAGTATTGC	CTGGGAAACACTCTGTGGACTG
mhcII	Major histocompatibility complex class IIb exon 2; pathogen recognizing protein of the adaptive immune response	(Lenz et al., 2009)	ENSGACT00000000425	GTCTTTAACTCCACGGAGCTGAAGG	ACTCACCGGACTTAGTCAG

Gene	Function	References	ENSEMBL ID	Forward primer	Reverse primer
Adaptive (continued)					
stat4	Signal transducer and activator of transcription 4; required for TH1-cell differentiation, opposes TH2 and TH17 like responses	(Kaplan, 2005; Premachandra et al., 2013; Wang and Secombes, 2013)	ENSGACT00000003538	CTCTCAGTTTCGAGGCTTGCTT	GGCAGTTGGCTCACATTGG
stat6	Signal transducer and activator of transcription 6; required for TH2-cell differentiation, regulates expression of TH2 relevant cytokine IL-4	(Robertson et al., 2015; Wang and Secombes, 2013)	ENSGACT00000011232	CTCAGCCACAGTTCCAACCGTTC	GTCGGATGTTCTGGACCTCGAGT
tcr-β	T-cell receptor β-chain; function in binding of MHC-peptide ligands to initiate adaptive immune response	(Smith-Garvin et al., 2009; Stutz et al., 2015; Yanagi et al., 1984)	ENSGACT00000016457	GAGGGCAAAAACTTCACCTG	TAGGAGAATCTGGCCGTTTG
tgf-β	Transforming growth factor β; cytokine with functions in cell growth, migration, differentiation and proliferation of T and B-cells	(Robertson et al., 2015; Zhu et al., 2013)	ENSGACT00000016968	TCCCGCTTCGTCACCAACCA	ACGTCTGTCTGGCCACATTCAC
Complement					
c7	Complement component 7; initializing function in the membrane attack complex of the complement system	(Brunner et al., 2017; Haase et al., 2014; Zhu et al., 2013)	ENSGACT00000009181	TGGCTCAAGCTCAGCACAACAG	AGCGACACGTGTTTGTTTGATCG
c9	Complement component 9; structural part of the membrane attack complex of the complement system	(Brunner et al., 2017; Haase et al., 2014; Zhu et al., 2013)	ENSGACT00000020968	CCGTGACGAACAAAGACTCAGTTG	TCTGACCGATGTCAGCACCTTG

Gene	Function	References	ENSEMBL ID	Forward primer	Reverse primer
Complement (continued)					
cfb	Complement factor B; activating complement component of the alternative pathway	(Brunner, 2016; Haase et al., 2014; Zhu et al., 2013)	ENSGACT00000027346	GAGCGTCGCACAATACAGGTTG	TACCACCGGAAGCGCACAAATC

References

Aerts-Toegaert, C., Heirman, C., Tuyaerts, S., Corthals, J., Aerts, J.L., Bonehill, A., Thielemans, K., and Breckpot, K. (2007). CD83 expression on dendritic cells and T cells: Correlation with effective immune responses. Eur. J. Immunol. *37*, 686–695.

Bedard, K., and Krause, K.-H. (2007). The NOX Family of ROS-Generating NADPH Oxidases: Physiology and Pathophysiology. Physiol. Rev. *87*, 245–313.

Birrer, S.C., Reusch, T.B.H., and Roth, O. (2012). Salinity change impairs pipefish immune defence. Fish Shellfish Immunol. *33*, 1238–1248.

Brunner, F.S. (2016). Parasitism along the speciation continuum - Variation of parasite effects across speciation stages of Gasterosteus aculeatus and their modification by environmental changes. Queen Mary University of London.

Brunner, F.S., Anaya-Rojas, J.M., Matthews, B., and Eizaguirre, C. (2017). Experimental evidence that parasites drive eco-evolutionary feedbacks. Proc. Natl. Acad. Sci. 201619147.

Calandra, T., and Roger, T. (2003). Macrophage migration inhibitory factor: a regulator of innate immunity. Nat. Rev. Immunol. *3*, 791–800.

Haarder, S., Kania, P.W., Bahlool, Q.Z.M., and Buchmann, K. (2013). Expression of immune relevant genes in rainbow trout following exposure to live Anisakis simplex larvae. Exp. Parasitol. *135*, 564–569.

Haase, D., Rieger, J.K., Witten, A., Stoll, M., Bornberg-Bauer, E., Kalbe, M., and Reusch, T.B.H. (2014). Specific Gene Expression Responses to Parasite Genotypes Reveal Redundancy of Innate Immunity in Vertebrates. PLoS ONE *9*, e108001.

Hibbeler, S., Scharsack, J.P., and Becker, S. (2008). Housekeeping genes for quantitative expression studies in the three-spined stickleback Gasterosteus aculeatus. BMC Mol. Biol. *9*, 18.

Huang, Y., Chain, F.J.J., Panchal, M., Eizaguirre, C., Kalbe, M., Lenz, T.L., Samonte, I.E., Stoll, M., Bornberg-Bauer, E., Reusch, T.B.H., et al. (2016). Transcriptome profiling of immune tissues reveals habitat-specific gene expression between lake and river sticklebacks. Mol. Ecol. n/a-n/a.

Kaplan, M.H. (2005). STAT4: A Critical Regulator of Inflammation In Vivo. Immunol. Res. *31*, 231–242.

Kasheta, M., Painter, C.A., Moore, F.E., Lobbardi, R., Bryll, A., Freiman, E., Stachura, D., Rogers, A.B., Houvras, Y., Langenau, D.M., et al. (2017). Identification and characterization of T reg–like cells in zebrafish. J. Exp. Med. *214*, 3519–3530.

Kissick, H.T., Dunn, L.K., Ghosh, S., Nechama, M., Kobzik, L., and Arredouani, M.S. (2014). The Scavenger Receptor MARCO Modulates TLR-Induced Responses in Dendritic Cells. PLoS ONE *9*, e104148.

Kovacevic, N., Hagen, M.O., Xie, J., and Belosevic, M. (2015). The analysis of the acute phase response during the course of Trypanosoma carassii infection in the goldfish (Carassius auratus L.). Dev. Comp. Immunol. *53*, 112–122.

Kraal, G., van der Laan, L.J.W., Elomaa, O., and Tryggvason, K. (2000). The macrophage receptor MARCO. Microbes Infect. *2*, 313–316.

Leemans, J.C., te Velde, A.A., Florquin, S., Bennink, R.J., de Bruin, K., van Lier, R.A.W., van der Poll, T., and Hamann, J. (2004). The Epidermal Growth Factor-Seven Transmembrane (EGF-TM7) Receptor CD97 Is Required for Neutrophil Migration and Host Defense. J. Immunol. *172*, 1125–1131.

Lenz, T.L., Eizaguirre, C., Becker, S., and Reusch, T.B. (2009). RSCA genotyping of MHC for high-throughput evolutionary studies in the model organism three-spined stickleback Gasterosteus aculeatus. BMC Evol. Biol. *9*, 57.

Marton, N., Baricza, E., &#xc9, Rsek, B., Buz&#xe1, S, E.I., Nagy, G., and rgy (2015). The Emerging and Diverse Roles of Src-Like Adaptor Proteins in Health and Disease. Mediators Inflamm. *2015*, e952536.

Maxson, J.E., Gotlib, J., Pollyea, D.A., Fleischman, A.G., Agarwal, A., Eide, C.A., Bottomly, D., Wilmot, B., McWeeney, S.K., Tognon, C.E., et al. (2013). Oncogenic CSF3R Mutations in Chronic Neutrophilic Leukemia and Atypical CML. N. Engl. J. Med. *368*, 1781–1790.

Mayumi, M., Takeda, Y., Hoshiko, M., Serada, K., Murata, M., Moritomo, T., Takizawa, F., Kobayashi, I., Araki, K., Nakanishi, T., et al. (2008). Characterization of teleost phagocyte NADPH oxidase: Molecular cloning and expression analysis of carp (Cyprinus carpio) phagocyte NADPH oxidase. Mol. Immunol. *45*, 1720–1731.

Premachandra, H.K.A., Elvitigala, D.A.S., Bathige, S.D.N.K., Whang, I., Lee, Y., De Zoysa, M., and Lee, J. (2013). Genomic structure and immunological response of an STAT4 family member from rock bream (Oplegnathus fasciatus). Fish Shellfish Immunol. *35*, 1829–1837.

Rao, D.N., and Naqvi, R.A. (2011). FoxP3: A Key Player in T Regulatory Biology. Indian J. Clin. Biochem. *26*, 1–2.

Rhodes, L.D., Wallis, S., and Demlow, S.E. (2009). Genes associated with an effective host response by Chinook salmon to Renibacterium salmoninarum. Dev. Comp. Immunol. *33*, 176–186.

Robertson, S., Bradley, J.E., and MacColl, A.D.C. (2015). Measuring the immune system of the three-spined stickleback – investigating natural variation by quantifying immune expression in the laboratory and the wild. Mol. Ecol. Resour. n/a-n/a.

Rønneseth, A., Ghebretnsae, D.B., Wergeland, H.I., and Haugland, G.T. (2015). Functional characterization of IgM+ B cells and adaptive immunity in lumpfish (Cyclopterus lumpus L.). Dev. Comp. Immunol. *52*, 132–143.

Shau, H., Gupta, R.K., and Golub, S.H. (1993). Identification of a Natural Killer Enhancing Factor (NKEF) from Human Erythroid Cells. Cell. Immunol. *147*, 1–11.

Smith-Garvin, J.E., Koretzky, G.A., and Jordan, M.S. (2009). T Cell Activation. Annu. Rev. Immunol. *27*, 591–619.

Stutz, W.E., Schmerer, M., Coates, J.L., and Bolnick, D.I. (2015). Among-lake reciprocal transplants induce convergent expression of immune genes in threespine stickleback. Mol. Ecol. *24*, 4629–4646.

Tabbara, I.A. (1993). Granulocyte colony-stimulating factor. South. Med. J. *86*, 350–355.

Wang, T., and Secombes, C.J. (2013). The cytokine networks of adaptive immunity in fish. Fish Shellfish Immunol. *35*, 1703–1718.

Wang, M.-H., Zhou, Y.-Q., and Chen, Y.-Q. (2002). Macrophage-Stimulating Protein and RON Receptor Tyrosine Kinase: Potential Regulators of Macrophage Inflammatory Activities. Scand. J. Immunol. *56*, 545–553.

Wen, Y., Shao, J.-Z., Xiang, L.-X., and Fang, W. (2006). Cloning, characterization and expression analysis of two Tetraodon nigroviridis interleukin-16 isoform genes. Comp. Biochem. Physiol. B Biochem. Mol. Biol. *144*, 159–166.

Yanagi, Y., Yoshikai, Y., Leggett, K., Clark, S.P., Aleksander, I., and Mak, T.W. (1984). A human T cell-specific cDNA clone encodes a protein having extensive homology to immunoglobulin chains. Nature *308*, 145–149.

Zhu, L., Nie, L., Zhu, G., Xiang, L., and Shao, J. (2013). Advances in research of fish immune-relevant genes: A comparative overview of innate and adaptive immunity in teleosts. Dev. Comp. Immunol. *39*, 39–62.
